# Peering Through the Polymer: Tracking Small Molecules to Improve Polymer Development

**DOI:** 10.1021/acs.macromol.5c03369

**Published:** 2026-05-13

**Authors:** Callum Johnson, Chloe M. Shilling, Matthieu Starck, William D. G. Brittain, Clare S. Mahon, Juan A. Aguilar

**Affiliations:** Department of Chemistry, Durham University, Lower Mount Joy, South Rd, Durham DH1 3LE, U.K.

## Abstract

Nuclear Magnetic Resonance (NMR) is a key tool in polymer research. It can quantify monomer uptake, track polymer degradation, and expose side reactions, additives, and impurities. Yet, much of this information, important for materials development, is hidden beneath the broad polymer signal. Here, we show how to cut through this barrier. We first rationalize established but often misunderstood relaxation methods (CPMG, PROJECT, WASTED), then introduce *T*_*2*_ *-filtered pure shift* and *DOSY* methods, and finally, demonstrate how simple processing can strip away the polymer signal in experiments such as COSY and HSQC. This toolkit will have broad applications in identifying unwanted species, optimizing reactions, and, in general, improving product development.

## Graphical abstract

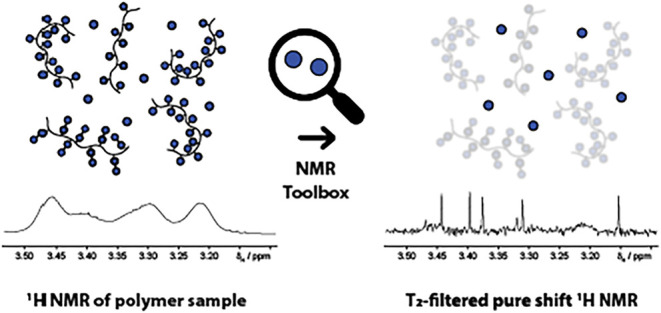

## Introduction

Synthetic polymers underpin modern life^1^ in fields as diverse as electronics,^2^ packaging,^3^ sensors,^4^ and biomedicine.^5^ While applications of Nuclear Magnetic Resonance (NMR) to the study of polymers are well established, the presence of small molecules in polymeric samples is sometimes overlooked, perhaps because their presence is sometimes not obvious (Figure 1a). The consequences and implications of the contaminants are varied, but it is important to detect them to avoid problems. For example, the presence of unreacted monomers or of monomers that have undergone side reactions represents an economic loss as this lowers the efficiency of the polymerization. Impurities may also change the type of product created,^6^ and even cause premature polymer termination;^7^ in addition impurities require time and resources to remove, and not removing them may violate regulatory dispositions, and cause health^8^ and environmental problems.^9^ Impurities can also impact the properties of the product (rheology, odors, thermal and mechanical stability, optical properties, etc.), and may even shorten the life of the product.^10^ Furthermore, some polymers release degradation products^11^ which can give rise to similar concerns. The source of the contaminants is, however, not limited to the polymerization; vendors use many additives^12^ and detecting theseis sometimes challenging.^13^ Detecting and characterizing small molecules is therefore important to diagnose problems and to optimize polymerizations.^14^

**Figure 1:**
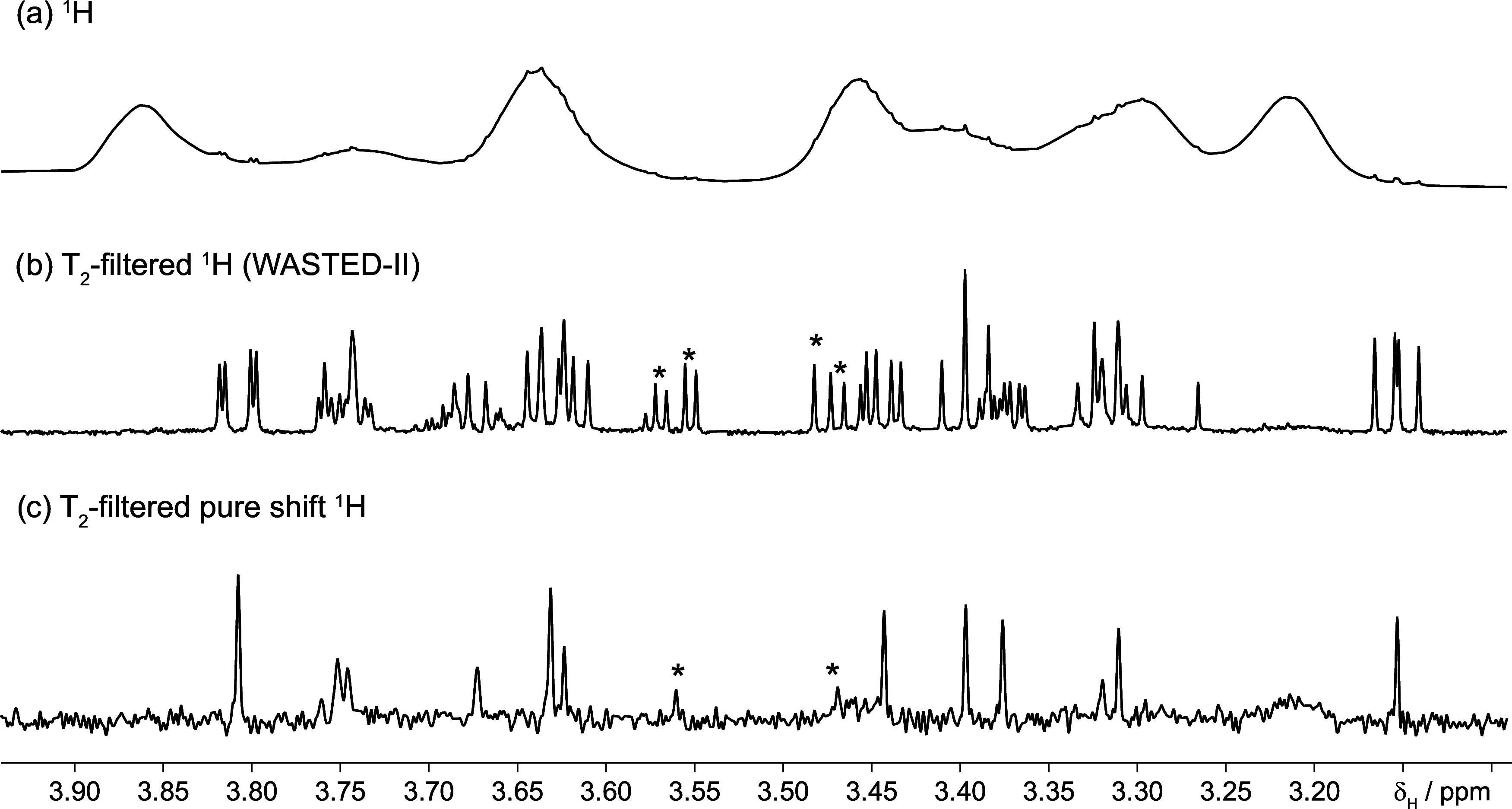
(a)­700 MHz ^1^H spectrum of the dialyzed output of Scheme 1 (**P1** + impurities). The spectrum is dominated by polymer signals, and it is impossible to assess the presence and number of signals due to small molecules. Attenuation of the polymer signal using a T_2_-filter (WASTED-II, (b)) reveals these signals, but counting peaks is difficult. T_2_-filtered pure shift NMR simplifies the spectrum (c) by collapsing multiplets into singlets and removes the polymer interference. It revealed 14 signals from three small molecules, one of which was unexpected (*). Some of the signals present after the T_2_ filters could be due to highly mobile parts of the polymer, but the T_2_-filtered DOSY (PROJECT-DOSY), see below, proves that this was not the case.

The problem is that the ^1^H NMR signals of these small molecules are often obscured by the resonances of the macromolecule, so that their presence in the material goes unnoticed (Figure 1a). The solution to the problem appears to be simple: suppress the polymer signal. However, while the concept is straightforward, existing solutions may not be easy to implement safely (long CPMG filters), or an existing solution has been overlooked or even forgotten (the use of apodization as relaxation filters), or no solution has been proposed (T_2_-filtered pure-shift experiments, for example). This publication reports a combination of NMR experiments that can be used to analyze the presence, number, and identity of small molecules in samples containing macromolecules. Importantly, these techniques are not power-demanding, and this reduces the risk of damaging sensitive electronic spectrometer components. The same techniques can be applied to the analysis of foods, metabolomic samples, and of biomedicines, but here we will focus on polymer samples. We have already demonstrated how one of these techniques (PROJECT) can be used to study the polymerization kinetics of 2-phenyl-2-oxazolines but the full toolkit is demonstrated here.^14^

The glycopolymer we used to demonstrate the potential of the toolkit consists of a poly­(acyl-hydrazide) scaffold to which we attached reducing sugars via formation of β-glycosides,^15,16^ as shown in Scheme 1 and in the Supporting Information. We purified the crude product of the reaction using dialysis. The result of a ^1^H NMR analysis was satisfactory at first sight (Figure 1a); however, there are weak, sharp signals on top of the broad ones. Sharp signals indicate the presence of small molecules, and in some cases, of signals from parts of the polymer with high mobility. We could have used chromatographic methods to isolate the small molecules, if any, but we instead used NMR techniques that provide answers without the need for physical separations, at least when the mixtures are not overly complicated. We have developed some of these techniques ourselves (see the author contributions section). Using these spectroscopic techniques, we wished to investigate:

**Scheme 1:**
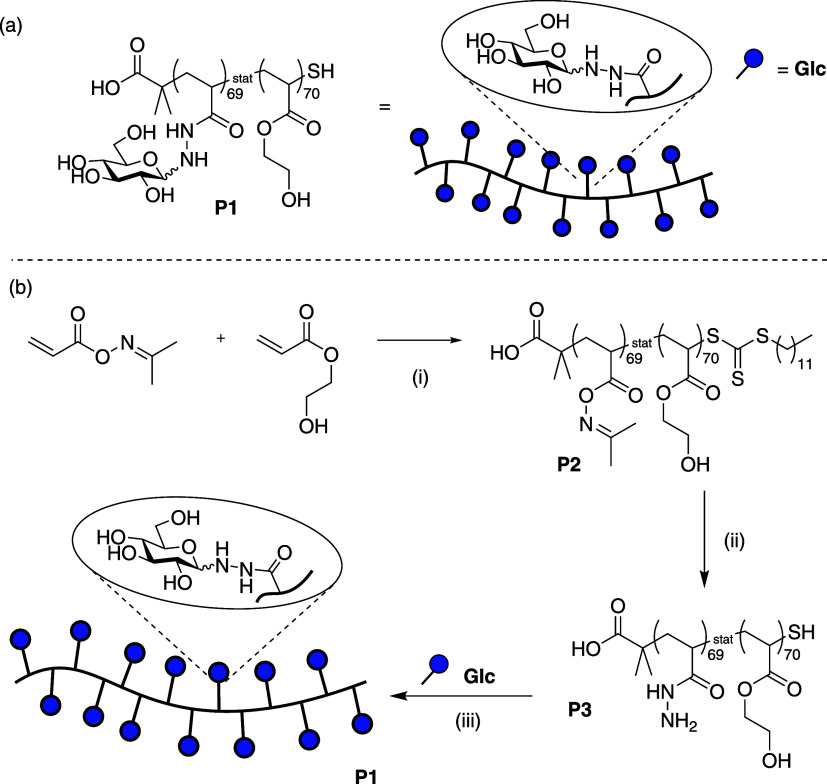
(a) Structure of Polymer **P1**. (b) Synthesis of **P1** (i) 2-(dodecylthiocarbonothioylthio)-2-methylpropionic acid, 2,2′-Azobis­(2-methylpropionitrile), 1,4-Dioxane, 70 °C, 1.5 h. (ii) N_2_H_4_.H_2_O, DMF, 0 °C, 1 h. (iii) 100 mM NaOAc/AcOH pH 4.5, 50 °C, 18 h

- whether the sample contained small molecules, for which we used PROJECT-based T_2_-filters (Figure 1b) and T_2_-filtered diffusiometry for which we developed a T_2_-filtered DOSY (PROJECT-DOSY, Figure 4).

- the number of contaminants and their approximate molecular size, for which we used PROJECT-DOSY and T_2_-filtered pure shift methods (Figures 1c, and 5).

- the structure of the contaminants, for which we used COSY and HSQC, from which we eliminated the polymer signal using simple processing methods, Figures 6 and 7.

## Does the Sample Contain Small Molecules?

Revealing the presence of small molecules can be achieved by removing the polymer signal by exploiting the differences in transverse relaxation (T_2_) between big and small molecules. Signals from big molecules decay faster in the time domain (short T_2_, broad signals) than signals from small molecules (long T_2_, sharp signals), although parts of the polymer with high mobility can also have long T_2_s. A simple filter can be implemented introducing a delay when magnetization is in the transverse plane. If the delay is long enough, signals from large molecules will drop below the noise level while signals from small molecules will be still detectable. These filters can produce filter times unattainable by other methods, however, they should not be used with experiments that produce phase-sensitive data if there is a phase sensitive alternative. This is because signals would be modulated according to the chemical shifts and couplings; however, they are useful when the data is already presented in magnitude mode, as we shall demonstrate. The alternative to these filters has been, until recently, the fast-refocusing Carr–Purcell–Meiboom–Gill (CPMG) pulse sequence.^17–19^ The problem with this approach is that fast refocusing requires a large amount of energy, and this limits the attainable filter time. While 80 to 150 ms filter times often suffice in metabolomics where these filters are often used, polymers may require much longer filters – between 600 and 1,240 ms in this case. Long and demanding CPMG filters are possible but are best avoided because the required large power deposition may damage the hardware (capacitors and possibly coils) or at least shorten its life span. This limit can be removed by using trains of perfect echoes instead of the spin–echoes trains of the CPMG pulse sequence.^20,21^ This is how the T_2_ filter called PROJECT works (*Periodic Refocusing of J-Evolution by Coherence Transfer*).^22^ While a common echo time in CPMG is 0.3 ms, the echo time may be as long as 10 ms in the case of PROJECT, thus lowering the power demands per unit of time−this is what allows PROJECT to achieve much larger filter times. A further benefit of PROJECT is that it is not field-dependent, while CPMG is.^23^ An alternative to PROJECT when a large water signal needs to be suppressed is a WASTED-I or -II pulse sequence, sequences that combine PROJECT and solvent signal suppression.^23^

The application of the WASTED-II filter to the glycopolymer sample, Figure 1b, revealed several contaminants. The presence of two anomeric signals (Figure SI-5) indicated the presence of two pyranose forms of glucose, but other species are likely to be present. Two glucose isomers should produce 12 signals (not counting the two anomeric signals), more if other species are present; unfortunately, it was difficult to count signals even after removing the polymer signal. We therefore decided to simplify the spectrum by turning all multiplets into singlets using pure shift NMR techniques. This approach also increases resolution as signals occupy less space.^24^ To achieve this, it was necessary to add T_2_-filtration to the pure shift pulse sequence. The next section describes how.

## How Many Signals Am I Seeing? T_2_-Filtered Pure Shift ^1^H NMR

There are many pure shift techniques, but the most efficient ones use the PSYCHE approach.^25^ Among these pulse sequences, we favor PSYCHE-TSE^26^ for its tolerance to miscalibrations and to strong coupling. PSYCHE-TSE offers inherent T_1_ and T_2_ filtration, but the inherent T_2_-filtration is insufficient when long filters are needed, and the T_1_ portion favors polymer signals. PSYCHE needs to be fitted with a proper T_2_-filter. It would be tempting to fit PSYCHE with a PROJECT module but there is a more efficient way; it is sufficient to add equal delays (d_3_/2) either side of the main J-refocusing PSYCHE pulse as shown in Figure 2. We can then reach long filters without worrying about the power deposition because no extra pulses are needed.

**Figure 2:**
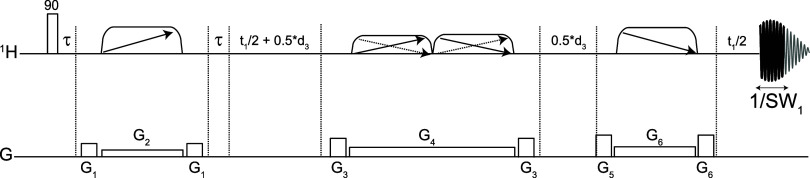
T_2_-filtered PSYCHE-TSE pulse sequence, derived from the original PSYCHE-TSE pulse sequence. The narrow rectangles denote a hard 90° pulses, the trapezoids with single arrows are 180° chirp pulses, and those with double arrows are low flip angle chirp pulses sweep frequency simultaneously in opposite directions. The filter time, d_3_, can reach arbitrary values without endangering the spectrometer, as no extra pulses are needed.

This pure shift experiment revealed 14 signals (not counting the anomerics, Figure 1c). We next sought to determine whether these signals could be attributed to highly mobile polymer segments, or to a molecule that interacts with the polymer and that for this reason escaped dialysis, or to a different molecule. This is the subject of the next section.

## Are These Signals from the Polymer or from Small Molecules?

Just because some signals survived a T_2_-filter it does not necessarily mean that they are from small molecules; they may belong to highly mobile parts of the polymer. NMR diffusiometry (Diffusion Ordered SpectroscopY, DOSY) can determine whether we are dealing with big or small entities. Of course, if we do not remove the polymer signals from the DOSY, the estimated diffusion coefficients will describe mostly the polymer, as seen in Figure 3, where we acquired a DOSY without T_2_-filtration.

**Figure 3:**
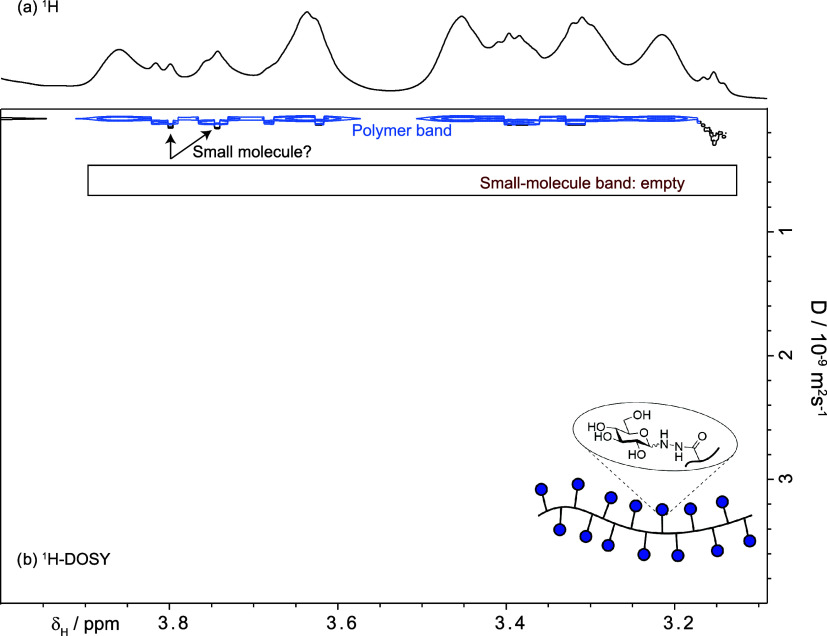
700 MHz ^1^H DOSY of the sample containing the polymer and the impurities. This indicated, incorrectly, that we had only species with an approximated mass of 141 kDa. The problem was that the polymer dominated the spectrum, so the resulting diffusion coefficients reflected the polymer diffusion coefficient. The presence of the small molecules is revealed in Figure 4.

To determine whether the signals that survived the T_2_-filter belong to the polymer or to small molecules, it is necessary to add a T_2_-filter to a DOSY. There are several options. We could have used the elegant PROJECTED^27^ pulse sequence that intertwines T_2_-filtration and diffusion encoding, but that is demanding for non-NMR experts; we could have used a CPMG-DOSY,^28^ but the problem is again that CPMG is not ideal for long filters. Notably, the large power deposition produced by the CPMG module can increase the temperature of the sample, change diffusion coefficients (and the apparent mass of the molecule inferred from them), and even trigger convection (anathema to diffusiometry). We decided therefore to design an alternative approach that is easier to use and less demanding on the instrument. We created a PROJECT-filtered DOSY (the pulse sequence can be found in the Supporting section). The main benefit of this approach is that the power deposition of the PROJECT module is much smaller than that of CPMG,^22^ and this is important for the present application, because the polymer signals must be attenuated below noise level to avoid underestimating the diffusion coefficient, and this requires T_2_-filters rarely attainable by CPMG.

The results of the T_2_-filtered DOSY (PROJECT-DOSY, Figure 4) demonstrate that

**Figure 4:**
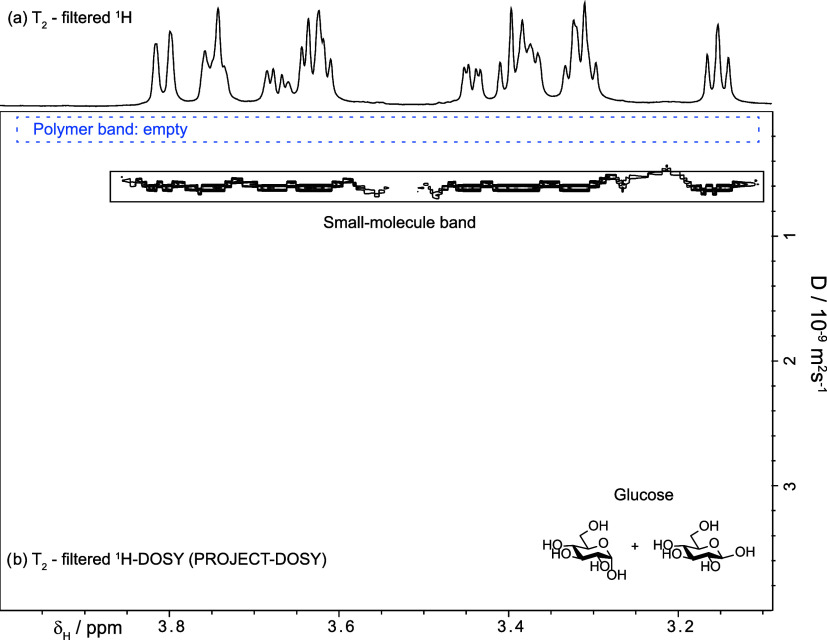
(a) 700 MHz ^1^H-WASTED-II spectrum. (b) PROJECT-DOSY removed the polymer signals and proved that the signals appearing in (a) are not due to highly mobile parts of the polymer; they were produced by small molecules. Compare with Figure 3.

- The signals that survived the T_2_-filter did not arise from highly mobile parts of the polymer; the diffusion coefficients are typical of small molecules. Compare Figures 3 and 4.

- Small molecules were still present after dialysis. A reversible interaction of a small molecule with the polymer would result in a reduced apparent diffusion coefficient (see below) − although viscosity can also alter diffusion coefficients − while a very strong interaction would have made their signals invisible after using the PROJECT filter.

- The diffusion coefficients were similar to those of pure glucose, albeit slightly smaller (6.6 × 10^–10^ m^2^ s^–1^ in the polymer-containing sample versus 6.1 × 10^–10^ m^2^ s^–1^ of pure glucose, SI-Section IV), either because the polymer increases the viscosity of the sample or because glucose interacts weakly with the polymer.

- In addition, all the molecules have the same diffusion coefficient and hence must be isomers or belong to a single but larger molecule. The possibility of a larger molecule was discarded after further spectroscopic investigation (see below).

- The third contaminant was too diluted to be detected under these conditions, but its nature (glycerol) is revealed in the next section.

A byproduct of the T_2_-filtered DOSY is that one can estimate the number of species with different volumes by counting diffusion bands, but only if the polymer signal is well suppressed and if there is no overlap between signals from species with different volumes, as this will create artificial diffusion bands.^29^

The presence of glucose anomers and, unexpectedly, glycerol was confirmed by analyzing signal multiplicities and their scalar connectivity. This is the subject of the next sections.

## Multiplicity Analysis. T_2_-Filtered J-Resolved Spectroscopy

NMR data analysis typically begins by examining signal multiplicity; however, this was difficult because multiplets in our spectra overlapped. We overcame this problem by collapsing multiplets into singlets (Figure 1c), but what was the multiplicity of those signals before being reduced to singlets? We used a T_2_-filtered J-resolved two-dimensional (2D) experiment^26^ to answer the question. This experiment produces a two-dimensional spectrum where the multiplicity of a signal can be analyzed in the indirect dimension (Figure 5 and SI-section IV); it produces pure shift spectra too as demonstrated in Figure 5b and in the Supporting section III. We achieved this by modifying the T_2_-filtered PSYCHE-TSE experiment of Figure 2 following Keeler and Morris’ prescriptions.^26,30^ The pulse sequence can be found in the Supporting section II. Using this experiment, we saw that the two signals suspected of being the third contaminant were doublets-of-doublets with a shared coupling of 11.9 Hz and two extra couplings. From these observations, we suspected the presence of glycerol. Glycerol has three signals, two of which with the multiplicity and coupling constants equal to those calculated (Figure 5). The third signal is too small to be detected, but we confirmed the presence of glycerol by comparing the sample’s J-resolved 2D experiment with a sample of glycerol in water (SI-section IV). We suspected that the source of glycerol was a spin-concentrator used during dialysis. We confirmed it by passing water through the spin concentrator and comparing its ^1^H NMR spectrum with the spectrum of a sample of pure glycerol in water. This shows how contaminants can go undetected if the right tools are not used.

**Figure 5:**
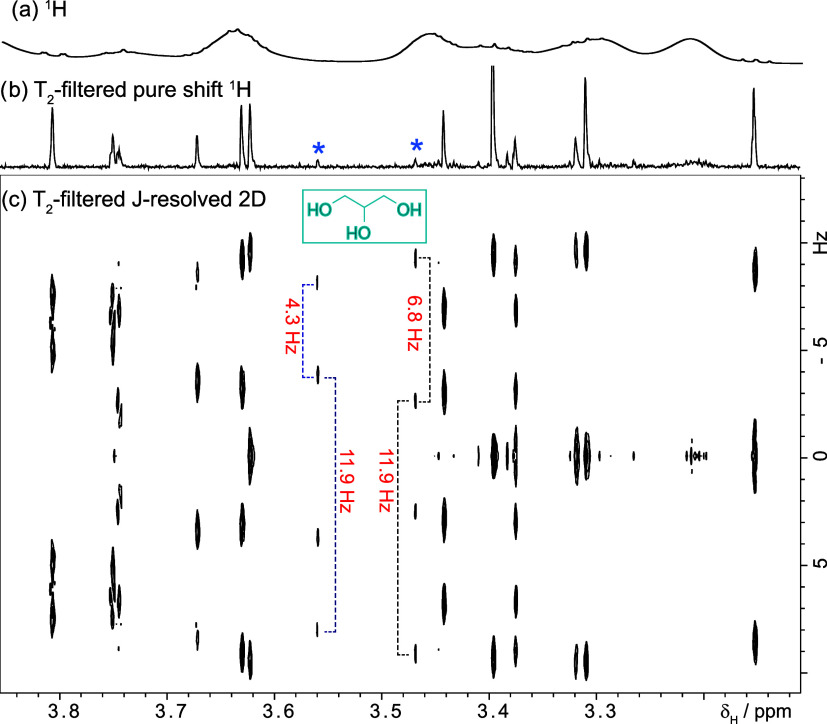
Determination of multiplicities using the new T_2_-filtered J-resolved experiment (T_2_-2DJ-PSYCHE-TSE). Signal multiplicity is analyzed in the indirect dimension where signals evolve only according to their couplings; for example, glycerol, a contaminant, produces the two doublets of doublets marked with a blue asterisk. (a) 700 MHz ^1^H spectrum of the polymerization output after dialysis. (b) Small-molecule signals selected with a T_2_ filter. (c) Polymer signals are absent in (b, c). The supporting section-III demonstrates how to obtain J-coupling and pure shift data from it.

The next step was to determine how the proton signals were connected. Could we prove that we had two glucose isomers, or that the glucose units further reacted? While the latter is unlikely, it is not unusual that degradation products react further. This is the subject of the next section.

## How Are The Proton Signals Connected? T_2_* - Filtered COSY

Homonuclear connectivity can be deduced using Correlated SpectroscopY (COSY) although it is still necessary to suppress the signals from the polymer. This has been done previously by adding a CPMG module to a COSY,^28^ however, there are solutions for magnitude-mode COSY that do not need a CPMG module, or any extra pulses for the matter. The simplest solution is to eliminate or attenuate the first points of the fid using apodization functions that are commonly used to process COSY.^31^ Figure 6b, d show two COSYs, one processed using sine-squared apodization (6d) and the other (6b) without it. The sine-square apodization used in (6d) attenuated the polymer signal enough so as not to interfere with the interpretation. The profile of the sine-square function is shown in Figure 6f. More attenuation can be achieved by changing the position and type of filter, as illustrated in the Supporting Information. This method has some advantages: (i) it may be applied to any COSY already acquired; (ii) it is simple, and almost all software can set these functions with little user intervention; (iii) it can reach arbitrary filter times without risking the spectrometer, as this is a processing method; however, care is necessary as unnecessarily long filters may result in unnecessary reductions in sensitivity. Worth noticing, too, is that these filters do not refocus field inhomogeneities (PROJECT, WASTED, and CPMG do) so that signal decay is controlled by T_2_* with the * indicating that field inhomogeneities contribute to T_2_.

The COSY obtained (Figure 6d) is consistent with glucose (Figure 6g).

**Figure 6:**
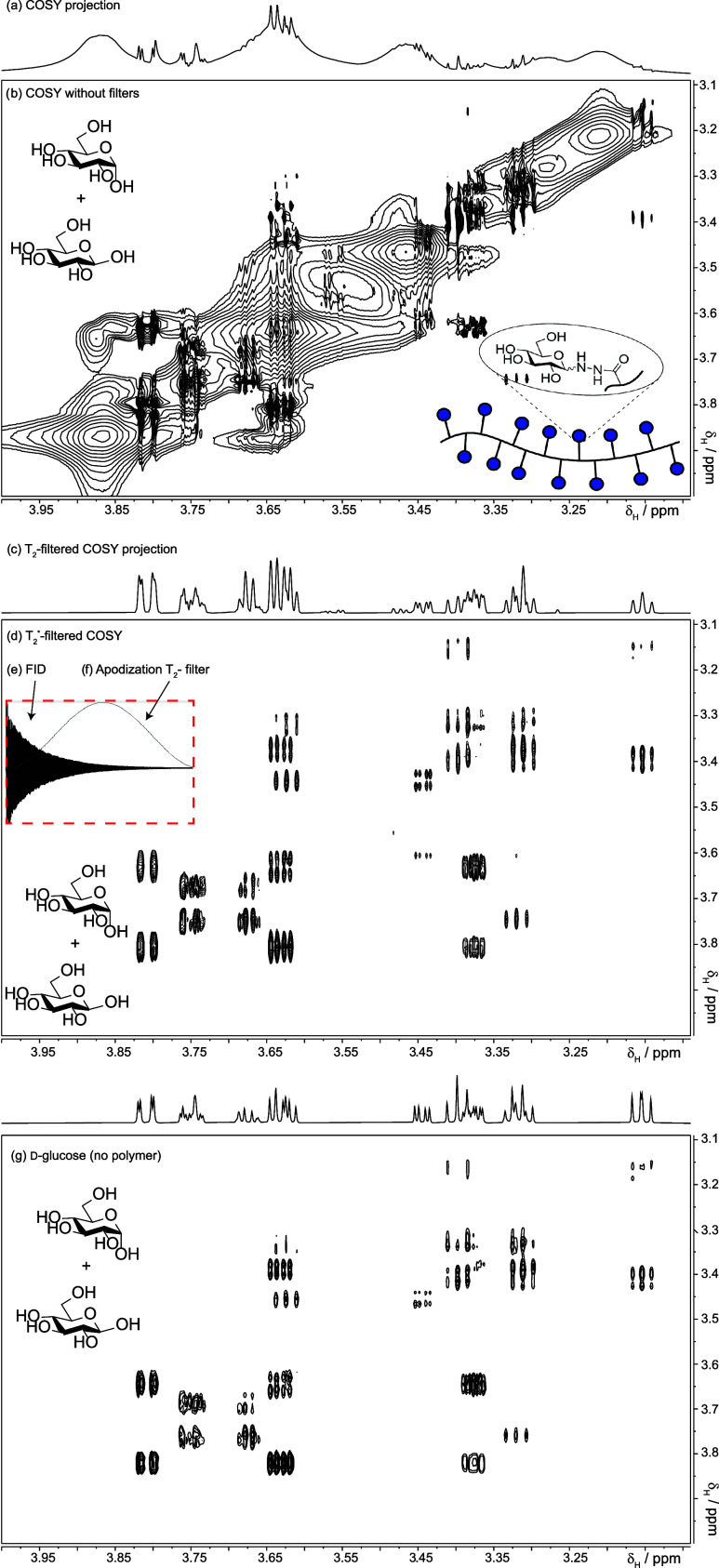
How to attenuate polymer signals in COSY. (b) 700 MHz ^1^H–^1^H COSY of the polymer sample in Scheme 1. Both polymer and contaminant signals are present, but the polymer obscures signals from the small molecules. Removing the polymer signal, as in (d), reveals the small-molecule signals and their coupling networks. The resulting spectrum matches the COSY of a reference sample of d-glucose (g). To obtain (d), we applied a simple mathematical filter (f) that suppresses the early part of the FID where polymer signals dominate. This procedure is explained in the Supporting section.

The following step was to connect the protons with their carbons. This is the subject of the next section.

## How Are Protons Connected to Their Carbons?

Although signal overlap is less problematic in the ^1^H–^13^C HSQC due to the greater signal dispersion, the presence of the polymer makes interpretation difficult, and in some cases impossible. In our case, it was difficult to distinguish the polymer from the contaminants (Figure 7b). The best solution is to remove the polymer signal from the HSQC by adding a T_2_ filter;^32^ however, we found a simple method that can be used with any HSQC already acquired, albeit at the price of losing the phase information. The method is the same processing method that we used to attenuate, even remove, broad signals from COSY, that is, using apodization to remove the early part of the fid, where broad signals are more prominent. The results must be displayed in magnitude mode, but discarding the phase information is tolerable in this case. The procedure is illustrated in the Supporting Information. In passing we note that backward linear prediction would be preferable to apodization to attenuate broad signals when processing phase sensitive data, but that most NMR users may find it more difficult to use. We suspected that glucose units were being released from the polymer backbone. This was confirmed by monitoring the sample over time using a WASTED-II filter (SI-Section-IV). The reaction progresses slowly and takes days to complete.

## Limitations and Precautions

Although the pure shift and apodization filters presented here can be extended to arbitrary durations without damaging the hardware (apodization is a processing method, and the T_2_-filtered pure shift only requires delays), determining their length requires careful consideration. The optimal filter length is dictated by the need to suppress polymer signals sufficiently so that they do not interfere with the experiment (as in the case of PROJECT-DOSY), or so that residual signals do not lead to misinterpretation (COSY or HSQC). Sensitivity is another critical factor: increasing the filter length results in greater attenuation of all signals, not only those originating from the polymer.

T_2_-filters are not quantitative because all signals (broad and sharp) are attenuated, and the degree of attenuation is a function of the signal’s T_2_ (for PROJECT and CPMG pulse sequences) or T_2_* (for apodization filters) because different signals may have different T_2_ values. In practice, this only affects PROJECT and WASTED-II (or CPMG) when they are used to derive kinetic information following the disappearance of the monomer signals, or while monitoring the appearance of degradation products. Any signal can be used to monitor changes, but to derive kinetic information it is important to check that T_2_ does not change significantly (T_2_ may increase with viscosity,^33^ and viscosity may increase when the polymerization progresses). This can be done by overlapping all spectra to ensure that the line width of the signals does not change significantly; however, the sample must be homogeneous and well shimmed, or the line width at half height would be 1/ πT_2_* instead of 1/ πT_2_ with the star signifying that it includes contribution to the line width due to field inhomogeneities.

To monitor polymerizations, minimizing significant changes in viscosity, we recommend carrying out the polymerization in a vessel, taking samples periodically, and diluting them with deuterated solvents before analyzing them.

Convection affects all the pure shift experiments described here, and all DOSY experiments based on a single stimulated echo, as is the case of PROJECT-DOSY. All low-viscosity solvents convect at all temperatures (high and low),^34^ while water does not usually convect at 25 °C. In our case, there was no measurable convection. We proved this by running a well-established convection test (SI-Section II). We took the precaution of leaving the sample in the magnet to reach the desired temperature to avoid changes in signal chemical shifts and to avoid triggering convection while the temperature equilibrates. For low-viscosity solvents, convection can be quenched by reducing the diameter of the NMR tube^35^ using 5 mm tubes with thick walls, or 3 mm tubes, or simply by adding glass rods^36^ to reduce the effective diameter.

NMR diffusiometry is a complex field that requires care and consideration, and polymer diffusiometry requires even more. While the subject is beyond the scope of this report, we would like to offer some advice. The main function of the T_2_-filtered DOSY is to prove that signals that survive the T_2_-filter are not from highly mobile parts of the polymer; however, it may be possible to estimate the mass of species from the diffusion coefficient,^37,38^ although, in the case of polymers, this yields an average mass for the different polymer species in the sample. In the case of the polymer that we were studying, we estimated an average 141 kDa mass in D_2_O, while gel permeation chromatography (GPC) in DMF indicated an *M*_n_ of 17.3 kDa for the parent scaffold **P2**, which would correspond to an approximate *M*_n_ of 28.6 kDa for **P1**. The GPC analysis of **P2** agrees with the estimated *M*_n_ of the polymer determined through conversion analysis using NMR spectroscopy. During the hydrazinolysis reaction used to remove oximes from **P2** (Scheme 1b­(ii)), it was expected that the majority of the terminal trithiocarbonyl linkages would also be cleaved, yielding a thiol as drawn. It is apparent, however, that a small proportion of this functionality remains intact, as observed in NMR spectra of **P3**/**P1** (Figures SI-3 and 4; small signal at 0.8 ppm). Polymer chains which bear the dodecyltrithiocarbonate group will be prone to self-assembly in aqueous medium on account of their amphiphilic character. This self-assembly will not occur in DMF, as it can solubilize both the hydrophobic dodecyl unit, and the hydrophilic remainder of the polymer chains. The aggregation of these chains in aqueous medium likely contributes to the discrepancy between apparent molecular weights estimated using NMR diffusiometry in D_2_O and GPC in DMF.

Variations in diffusion coefficients can also be produced by changes in viscosity, and aggregation, among other factors. Changes in viscosities are sometimes monitored using “spy” molecules of known diffusion coefficients, but this requires knowing whether the spy molecule interacts with the polymer. In general, it is better to be cautious or to not estimate the mass of the polymer using diffusion coefficients if one is not sure whether these effects play an important role. In the case of small molecules contained in polymer samples, the diffusion coefficient can change if the polymer introduces viscosity changes that cannot be corrected for by using “spy molecules” or if the small molecules interact with the polymer. Knowledge of this effect can sometimes cause further complications.

## Concluding Remarks

We have developed a set of NMR pulse sequences that can uncover small molecules hidden in polymer samples, their presence, number, and identity. We began by rationalizing established methods (CPMG, PROJECT, WASTED), then introduced new T_2_-filtered pure shift and diffusion techniques (PROJECT-DOSY), and finally showed how broad signals can easily be removed from COSY and HSQC using simple processing methods. The new pure shift and multidimensional experiments offer unlimited filter times—a crucial advance, since different parts of a polymer often demand filtration beyond what most current techniques can handle safely.

We have used the information gathered in this publication to design more stable polymers and to deal with the spin-concentrator contaminant. We hope that the information derived from these techniques will help chemists analyze their polymer samples and ultimately produce more competitive products.

## Experimental Section

All spectra reported in the manuscript were acquired with a 700 MHz Bruker spectrometer equipped with a BBO Prodigy cold probe in D_2_O at 25 °C (unless otherwise stated). The probe can deliver a maximum gradient field of 53 G cm^–1^. All the experiments were run using the same sample. The sample was prepared as follows:

We dissolved 10 mg of the purified polymer (see SI-section-I) in 600 μL of D_2_O. We did not modify the pD after dissolving the sample. We used a 5 mm NMR tube. The estimated glucose loading was 75%. The sample was introduced in the spectrometer and left undisturbed for 15 min for the temperature to equilibrate.

Other NMR experiments that we used to characterize the synthetic procedure were acquired using a 400 MHz spectrometer (see SI, section I).

The spectrum of Figure 1b was obtained using the WASTED-II pulse sequence (this combines the PROJECT module, with a solvent suppression module). The total filter time was 0.96 s, individual spin–echo times took 10 ms. This required 48 perfect echoes. The repetition time was 2.5 s of which 1.5 s comprised the acquisition. Thirty-two transients were acquired in 15 min, and 28 s. Eight steady state (dummy) transients were used. The spectral window covered 10.5 kHz and was centered on the HDO peak. The position of the extra suppression peaks was pushed to 3.5 kHz from the center of the spectrum.

Convection was ruled out running the convection test described in the SI–Section II.

The T_2_-filtered pure shift spectrum of Figure 1c was produced using the pulse sequence shown in Figure 2 (the code of the pulse sequence can be obtained from the Supporting section). A filter time (d_3_) of 600 ms was used. The spectral widths of the F2 and F1 dimensions were 8620 and 53.9 Hz, respectively. The duration of the 10 kHz saltire pulse was 30 ms long. Thirty-two real increments were acquired each using 48 transients. The repetition time was 2 s of which 1 s comprised the acquisition time. The experimental time was 1 h and 16 min, although the time required can be just under 15 min if the contaminants are not as diluted as in this case.

The T_2_-filtered J-resolved experiment of Figure 5 was produced using a 2DJ version of the pulse sequence of Figure 2 adapted as prescribed by Keeler and Morris (the pulse sequence code can be seen in the Supporting section). A filter time (d_3_) of 600 ms was used. The spectral widths of the F2 and F1 dimensions were 3.9 kHz and 50.0 Hz, respectively. The duration of the 10 kHz saltire pulse was 30 ms long. Sixty-four complex increments were acquired each using 16 transients. The repetition time was 2.1 s of which 1.1 compressed the acquisition time. The experimental time was 1 h and 11 min.

The DOSY plot of Figure 3 was produced using the Bruker pulse program *ledbpgp2s* (an echo-stimulated pulse sequence fitted with bipolar pulse gradients and a longitudinal eddy current delay module). Ten gradient amplitudes ranging from 1.016 to 40.66 G cm^–1^ spaced in equal steps of gradient squared were used. Sixteen transients were collected. Four steady state transients were used. The number of complex data points was 27,396. The spectral window covered 6.8 kHz. The diffusion-encoding pulsed gradient duration (δ, p30*2) was 2.0 ms. The diffusion time was (Δ, d20) 300 ms. The gradient stabilization delay was 0.2 ms. The Longitudinal Eddy Current Delay (LED) time (d21) was 5 ms. The repetition time was 7.0 s of which 2.0 s comprised the acquisition time. The results were analyzed using TopSpin 4.4 and a monoexponential fittings. The experimental time was 23 min.

The PROJECT-DOSY of Figure 4 was produced the pulse sequence deposited in the Supporting section. The PROJECT filter time was used 1.28 s. This long filter was necessary to ensure that residual polymer signals were too small to interfere with the estimation of the diffusion coefficients. This required 64 perfect echoes with individual spin echoes taking 10 ms. Ten gradient amplitudes ranging from 0.981 to 39.233 G cm^–1^ spaced in equal steps of gradient squared were used. Sixteen transients were collected. Four steady state transients were used. The number of complex data points was 27,396. The spectral window covered 6.8 kHz. The diffusion-encoding pulsed gradient duration (δ, p30*2) was 1.2 ms. The diffusion time was (Δ, d20) 250 ms. The gradient stabilization delay was 0.2 ms. The Longitudinal Eddy Current Delay (LED) time (d21) was 5 ms. The repetition time was 7.0 s, of which 2.0 s comprised the acquisition time. The results were analyzed using TopSpin 4.4 and a monoexponential fittings. The experimental time was 23 min.

The COSY of Figure 6 was recorded using a 90° - t1–90° pulse sequence fitted with pulsed field gradients (the Bruker *cosygpppqf* pulse sequence). We inverted the polarity of the first field gradient to minimize t1-noise.^39^ The spectral windows spanned 4.5 kHz. Five-hundred and 12 real increments were used, each comprising two transients. The repetition time was 1.4 s of which 0.6 s comprised the acquisition time. A short double spinlock (SLx–2SLy) was used after each transient to eliminate rapid pulsing artifacts. The experimental time was 27 min. The processing method is described in the Supporting section.

The multiplicity edited HSQC of Figure 7 was produced using the Bruker pulse sequence *hsqcedetgpsp.3*. The spectral windows (^1^H and ^13^C) covered 4.5 and 13 kHz, respectively. Two transients and 512 real increments were used. The experiment was optimized for a J_HC_ of 145 Hz. The repetition time was 1.2 s, of which 0.2 s comprised the acquisition time. The experiment was processed as described in the Supporting section.

**Figure 7:**
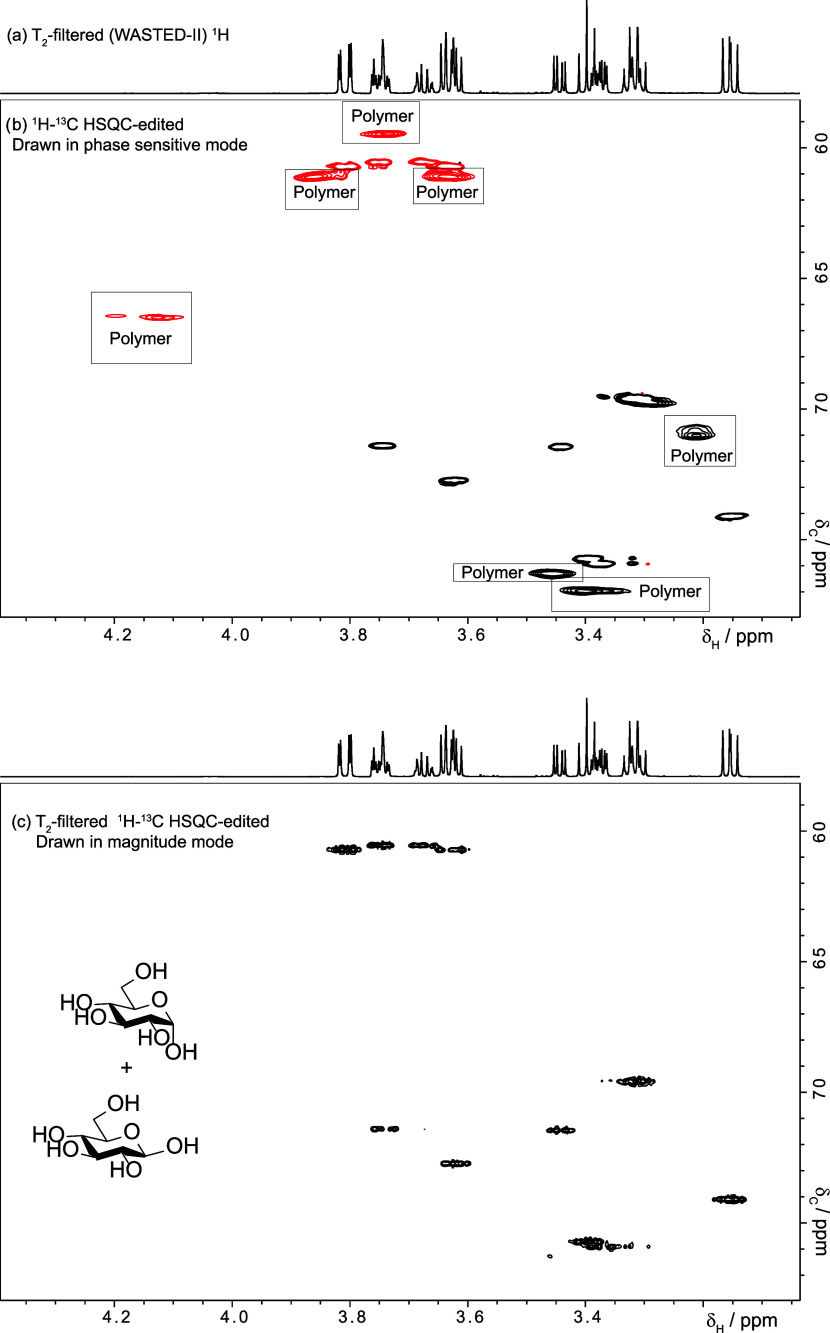
How to remove broad (polymer) signals from a ^1^H–^13^C HSQC. (b) 700 MHz, ^1^H–^13^C multiplicity edited HSQC of the sample shown in Scheme 1. While polymer signals hinder identification of small molecules in (b), their removal in (c) reveals the contaminant to be a mixture of d-glucose anomers (c). For clarity, the proton spectrum in (a) was replaced with a polymer-free version to aid assignment (WASTED-II). The ^1^H–^13^C fingerprint in (c) matches that of glucose. The color (phase) difference between (b) and (c) arises from the use of phase-sensitive versus magnitude displays. Glycerol was not detected under the 40 min acquisition time of the HSQC.

^1^H, WASTED-II, and T_2_-filtered pure shift experiments (PSYCHE-TSE and J-resolved PSYCHE) should be phased, while the apodization-based COSY and HSQC should be processed in magnitude.

Examples of each experiment/pulse sequence can be found here: DOI: http://doi.org/10.15128/r1jq085k08p

## Supplementary Material



## Notes

Synthetic procedures, pulse sequences, processing methods, as well as DOSY plots (PDF)

## Abbreviations

CPMG: Carr–Purcell–Meiboom–Gill
COSY: COrrelated SpectroscopY
DOSY: Diffusion Ordered SpectroscopY
FID: free-induction decay
GPC: Gel Permeation Chromatography
HMBC: Heteronuclear Multiple Bond Correlation
HSQC: Heteronuclear Single Quantum Correlation
LED: Longitudinal Eddy Current Delay
NMR: Nuclear Magnetic Resonance
PFG: pulsed field gradients
PROJECT: Periodic Refocusing of JEvolution by Coherence Transfer
PSYCHE: Pure Shift Yielded by Chirp Excitation
rf: radiofrequency
T1: longitudinal relaxation time constant
T_2_: transverse relaxation-time constant without contributions from field inhomogeneities
T_2^*^_: transverse relaxation time-constant in the presence of field inhomogeneities (nonperfect shims, for example)
WASTED: Water Suppression with a Transverse relaxation filter that Eliminates Distortions. While we have the term J-resolved experiment, it is common to refer to this experiment as to 2DJ
