# Supplementary material for: Peering Through the Polymer: Tracking Small Molecules to Improve Polymer Development
Source: Macromolecules. 2026 May 13;59(10):6077–87. doi: 10.1021/acs.macromol.5c03369 (PMC13217616; doi:10.1021/acs.macromol.5c03369)
Supplement: Supplementary file 1 [file ma5c03369_si_001.pdf]

Peering Through the Polymer: Tracking Small Molecules to Improve Polymer Development

Callum Johnson[1], Chloe M. Shilling, [1] Matthieu Starck, [1] William D. G. Brittain, [1] Clare S. Mahon, [1] and Juan A. Aguilar[*1]

AUTHOR ADDRESS: Department of Chemistry. Durham University. Lower Mount Joy, South Rd, Durham, DH1 3LE, UK

KEYWORDS: NMR, polymers, relaxation filters, T_2-filtered pure shift NMR, T_2-filtered DOSY.

Corresponding author: j.a.aguilar@durham.ac.uk.

SECTION I: Preparing the polymer.

Synthetic methods.

Full ^1H and ^1H-WASTED-II data Error! Reference source not found.

SECTION II: Pulse sequences

WASTED-II.

PROJECT-SL.

T_2-filtered PSYCHE-TSE and the macro necessary to process it.

T_2-filtered J-resolved PSYCHE-TSE.

PROJECT-DOSY.

Convection-test.

SECTION III: Tutorials

How use apodization to attenuate broad signals in a magnitude mode COSY. Topspin and MestreNova examples.

How use apodization to attenuate broad signals in a HSQC using Topspin or MestreNova.

How to process PROJECT-DOSY.

SECTION IV: additional evidence

Comparing PROJECT-DOSY results obtained using a sample of glucose in D_2O and a sample with just glucose.

Comparison between the T_2-filtered J-Resolved PSYCHE-TSE of glycerol dissolved in D_2O with glycerol in the polymer sample.

Comparison between ^1H-NMR of glycerol dissolved in D_2O with glycerol in a water sample passed through the spin concentrator.

SECTION I: Synthesis and characterization of the polymer

General Details

Dialysis membrane (regenerated cellulose with a 3.5 kDa molecular weight cut-off) was obtained from Spectrum Dialysis. Spin concentrators were purchased from Merck Millipore (molecular weight cut off 10 kDa) RAFT chain transfer agent *S*-1-dodecyl-*S*'-(α,α'-dimethyl-α''-acetic acid)trithiocarbonate (DDMAT) was synthesised according to a literature method. [1] All other chemicals were synthesised or purchased from Sigma Aldrich, Fischer Scientific or Fluorochem and used as received unless otherwise stated. Hydroxyethyl acrylate (HEA) (10 mL) was dissolved in H_2O (60 mL) and washed with hexane (4 × 60 mL). The aqueous layer was saturated with NaCl and HEA extracted into EtOAc (60 mL), dried with sodium sulfate and evaporated to dryness. HEA was passed though basic alumina immediately prior to use.

Instruments

NMR spectra acquired during synthesis and initial characterisation of polymers were recorded on a Bruker DRX spectrometer operating at 400.13 MHz for ^1H, using commercially available deuterated solvents ($CDCl_3$ (δH = 7.26 ppm) and D_2O (δH = 4.79 ppm)).

All other spectra were collected using a Bruker spectrometer operating at 699.94 MHz for ^1H. The spectrometer was equipped with a BBO Prodigy probe.

Gel permeation chromatography measurements were conducted using an Agilent 1260 instrument equipped with differential refractive index detector and a pair of PL gel 5 µm Mixed-D columns (300 × 7.5 mm) with a guard column (Polymer Laboratories Inc.) connected in series. Chromatography was performed in DMF containing 1.0 g/L LiBr (0.6 mL/min) at 50 °C.

Synthesis & Characterisation

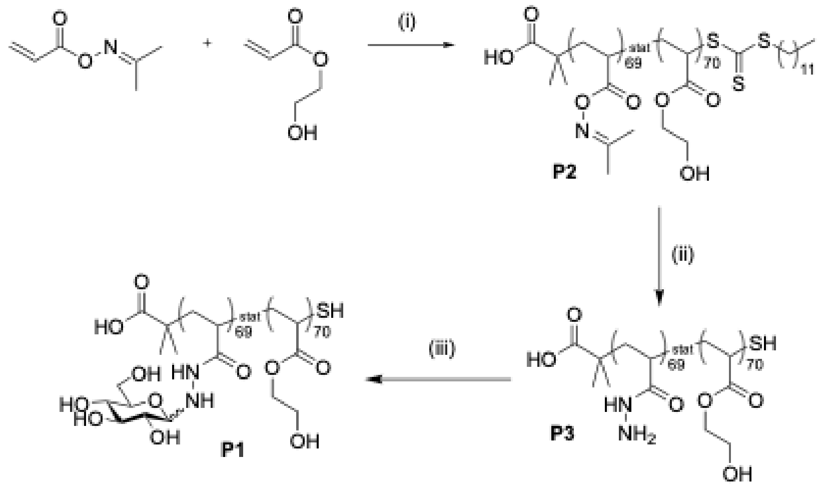

Scheme S1 Synthesis of **P1** (i) 2,2'-azobis(2-methylpropionitrile), 1,4-dioxane, 70 °C, 1.5 h. (ii) $N_2H_4.H_2O$, DMF, 0 °C, 1 h. (iii) 100 mM NaOAc/AcOH pH 4.5, 50 °C, 18 h.

Acetone oxime acrylate[2]

Procedure adapted from literature.[2] Acetone oxime (8.10 g, 110 mmol) and triethylamine (11.2 g, 110 mmol) were dissolved in chloroform (80 mL) and cooled to 0 °C. Acryloyl chloride (8.70 g, 110 mmol) in chloroform (40 mL) was added dropwise to the reaction mixture, which was stirred for 3 h at room temperature. The reaction mixture was then washed with water (3 × 80 mL) and saturated $NaHCO_3$ solution (3 × 80 mL) before being dried over Na_2SO_4. The solvent was removed *in vacuo* to afford acetone oxime acrylate as a pale-yellow oil (3.4 g, 24%). δ_H (400 MHz, $CDCl_3$) 2.07 (6 H, d, C(C**H**$_3$)$_2$), 5.93 (1 H, dd, C**H**), 6.22 (1 H, dd, C**H**), 6.54 (1 H, dd, C**H**). δ_C (101 MHz, $CDCl_3$) 17.00, 22.04, 126.68, 131.70, 163.69, 164.42. *m/z* (ESI) 128.15 ($(M+H)^+$, 100%). All data is consistent with that previously reported in literature.

*Poly(acetone oxime acrylate-stat-hydroxyethyl acrylate) (**P2**)*

2,2'-azobis(2-methylpropionitrile) (AIBN) (3.3 mg, 0.020 mmol), DDMAT (36.5 mg, 0.10 mmol), acetone oxime acrylate (1.14 g, 9.0 mmol) and HEA (1.04 g, 9.0 mmol) were combined in 1,4-dioxane (2.0 mL) and deoxygenated by sparging with argon for 20 min. The solution was then stirred in a preheated oil bath at 70 °C. After 1.5 h, the polymerisation was quenched by rapid cooling in $N_{2(l)}$ and exposure to air. The polymer was purified by dropwise addition into cold Et_2O, redissolved in CH_2Cl_2 and the precipitation repeated twice before drying *in vacuo* to yield **P2** as a yellow-white solid (0.81 g). δ_H (400 MHz, $CDCl_3$) 0.90 (3H, t, ω-C***H$_3$***), 1.28 (br, (C***H$_2$***)$_{11}$), 1.5-2.2 (br, polymer backbone), 2.02 (br, C(C***H$_3$***)$_2$), 2.07 (br, C(C***H$_3$***)$_2$), 3.65-3.87 (br, OCH_2C***H$_2$***OH), 4.04-4.36 (br, OC***H$_2$***CH_2OH).

Table 1 Characterisation data for polymer scaffold **P2**. [a]As determined by conversion analysis using ^{1}H NMR spectroscopy. [b]As determined by gel permeation chromatography in DMF containing 1 g/L LiBr at 50 ºC (0.6 mL min^{-1}), calibrated against near monodisperse poly(methyl methacrylate) standards.

Polymer	M1 / eq.	Con. / %	HEA / eq.	Con. / %	M_n[a]	M_n[b]	M_w[b]	Đ
**P1**	90	78	90	77	17300	18500	23900	1.29

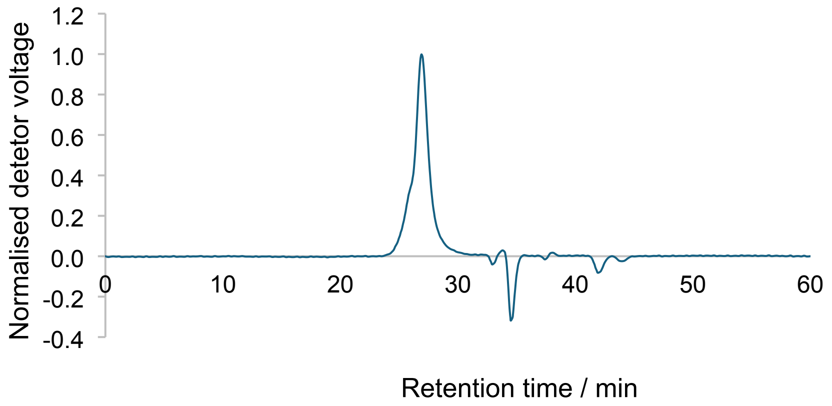

Figure S1. Differential refractive index gel permeation chromatogram of **P2**, acquired using DMF containing 1.0 g/L LiBr (0.6 mL/min) at 50 °C.

*Poly(acryloyl hydrazide-stat-hydroxyethyl acrylate) (**P3**)*

Hydrazine hydrate (300 µL, 9.4 mmol) was added to a solution of **P2** (100 mg, 5.8 µmol) in DMF (0.5 mL) at 0 °C and stirred for 1 h. The solvent was decanted, leaving a white precipitate which was dissolved in water and dialysed against water. The product was isolated by lyophilisation to afford **P2** as a white solid (45 mg, 54%). δ_H (400 MHz, D_2O) 1.3-2.2 (br, polymer backbone), 3.70-3.82 (br, $OCH_2C\boldsymbol{H_2}OH$), 4.04-4.22 (br, $OC\boldsymbol{H_2}CH_2OH$).

P1

P3 (8 mg, 0.55 µmol) was dissolved in a solution of sodium acetate buffer (0.1 mL, 100 mM, pH 4.5) and aniline (1% v/v). D-glucose (13 mg, 75 µmol) was added before the mixture was stirred at 50 °C for 18 h. The reaction mixture was concentrated by centrifugation (3845 *g*, 40 min; M_w cutoff 3.5 kDa), diluted with water and concentrated twice more (3845 *g*, 30 min) before lyophilisation to yield **P1** as a white solid (7.2 mg, 59%; extent of conjugation 75%).

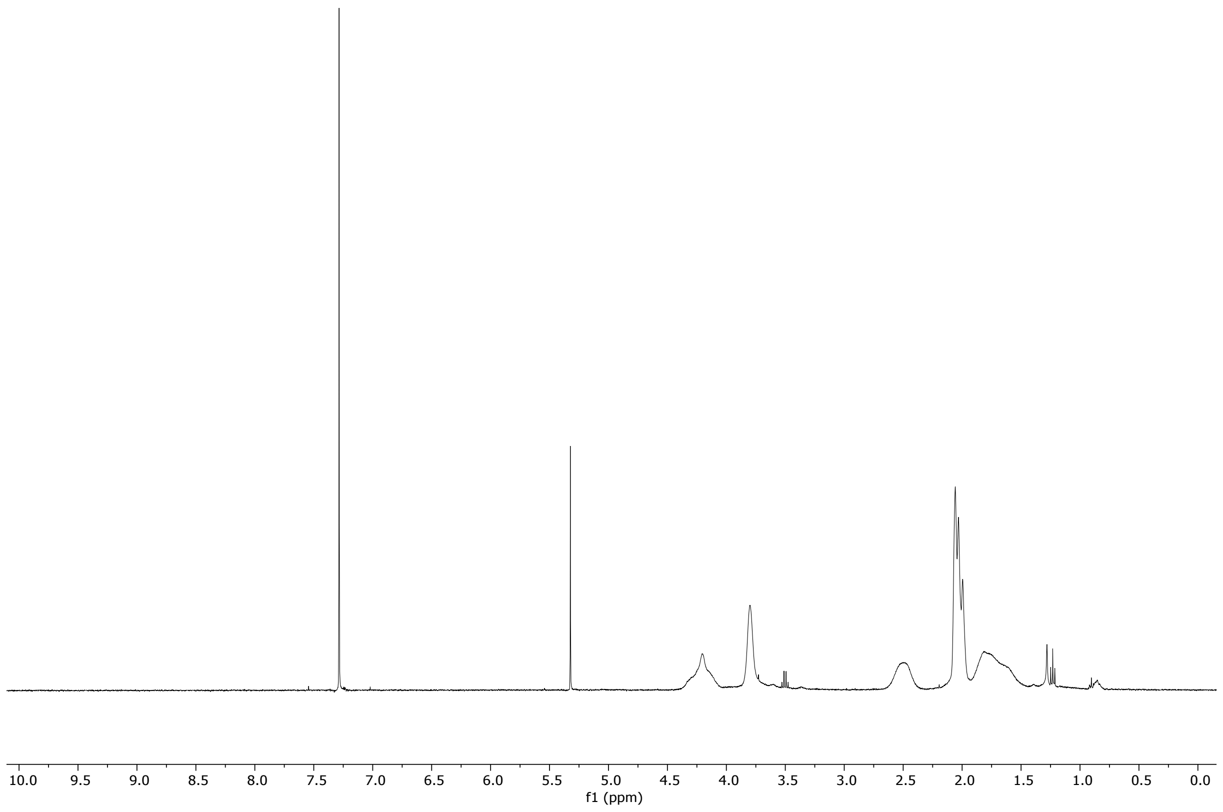

Figure S2.^{1}H NMR spectrum (400 MHz, $CDCl_3$) of **P2**.

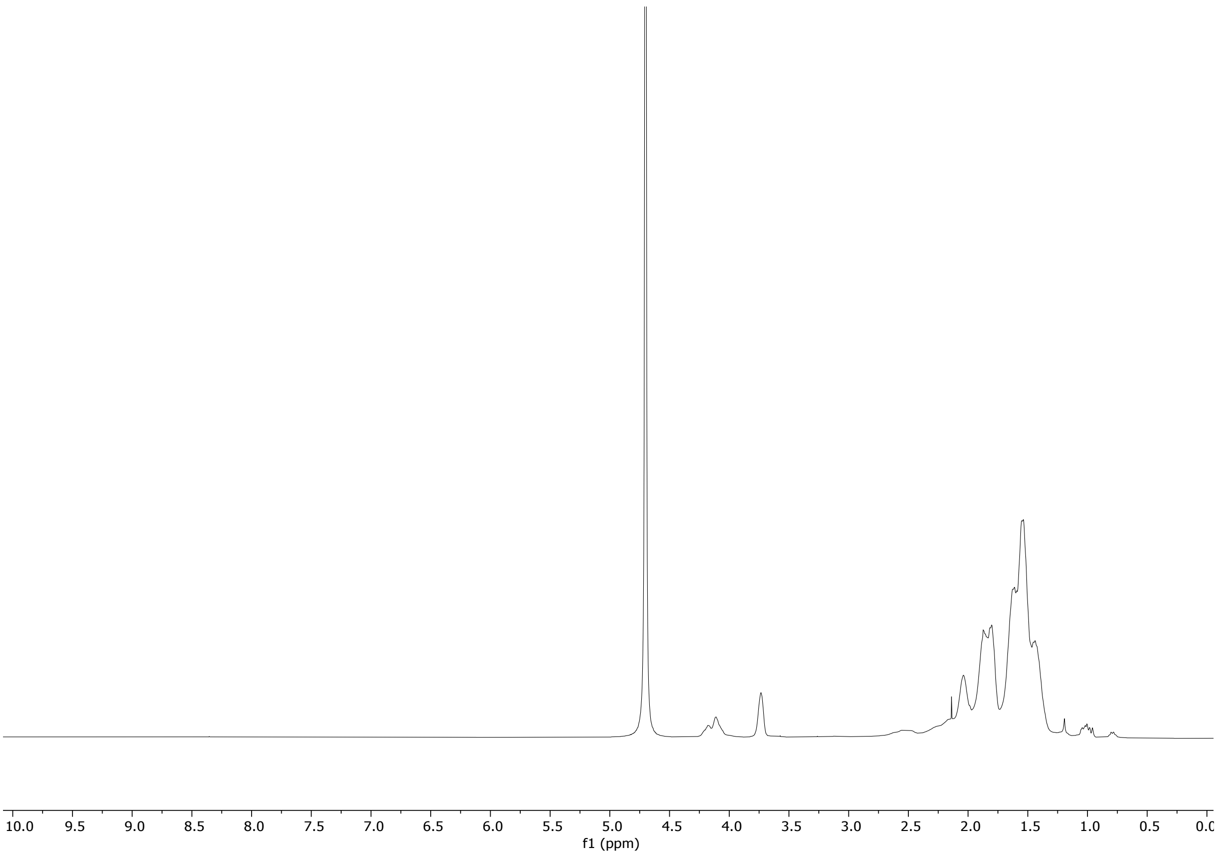

Figure S3.^{1}H NMR spectrum (400 MHz, D_2O) of **P3**.

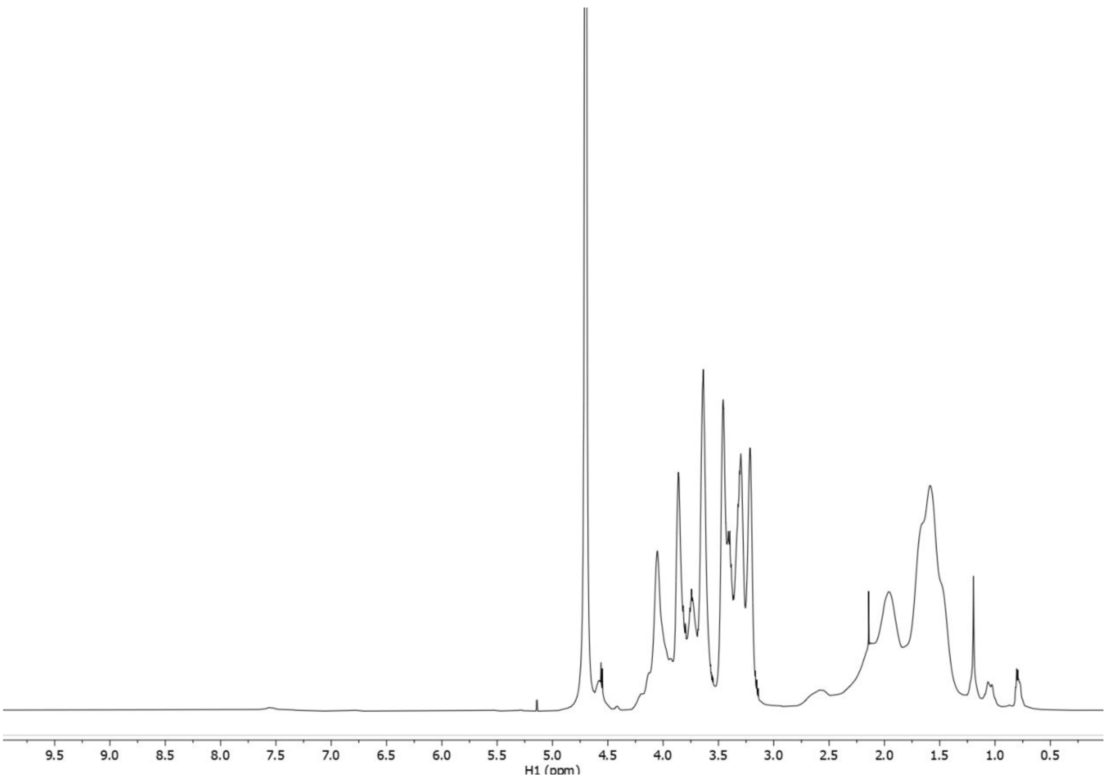

Figure S4.^{1}H NMR spectrum (700 MHz, D_2O) of **P1**.

^{1}H and ^{1}H-WASTED-II data.

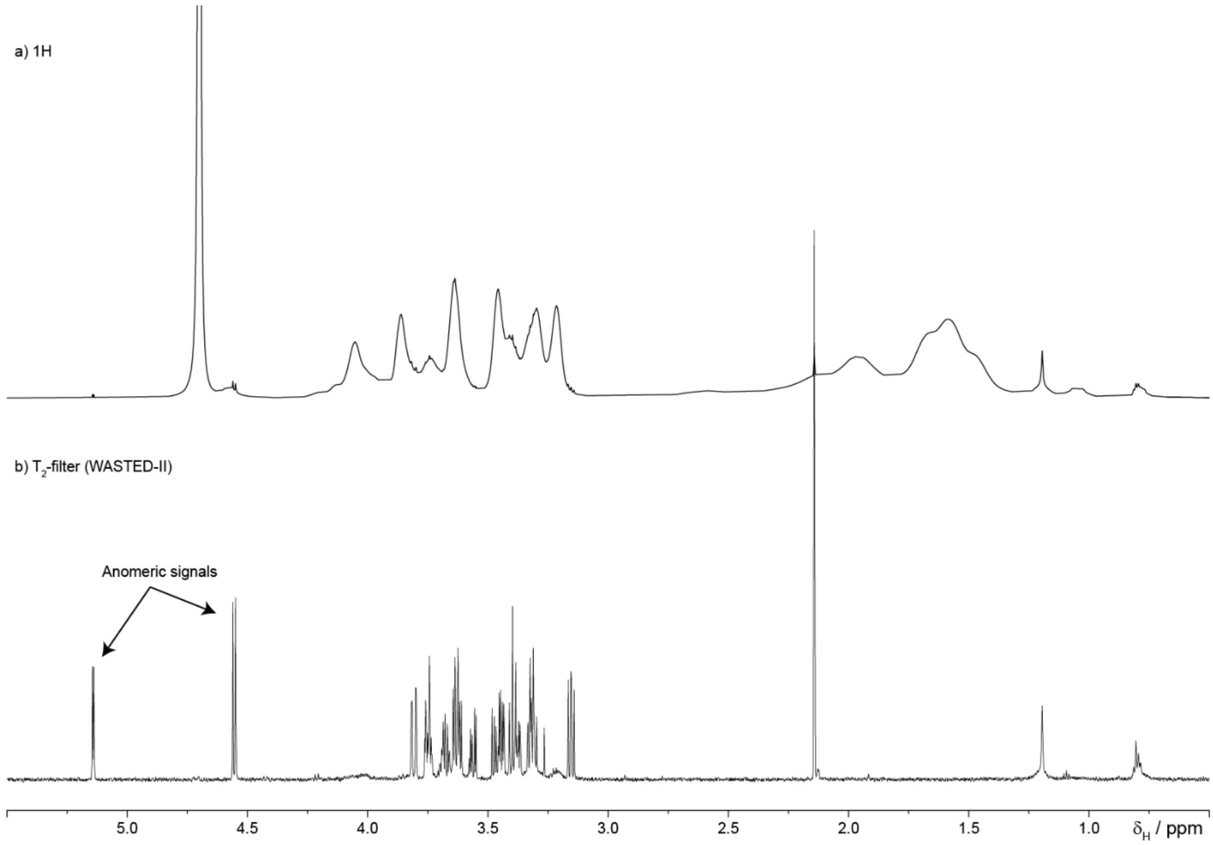

Figure S5. a) 700 MHz ^{1}H spectrum of the polymerization output after spin-concentration (dialysis). b) Same but using a WASTED-II pulse sequence. WASTED-II is a combination of PROJECT (T_2-filtration) and Robust-5 (suppression of the water signal). The solvent was D_2O and the temperature 25 °C.

SECTION II: Pulse sequences

WASTED-II.

PROJECT-SL.

T_2-filtered PSYCHE-TSE and the macro necessary to process it.

T_2-filtered J-resolved PSYCHE-TSE.

PROJECT-DOSY.

Convection-test.

The WASTED-II pulse sequence

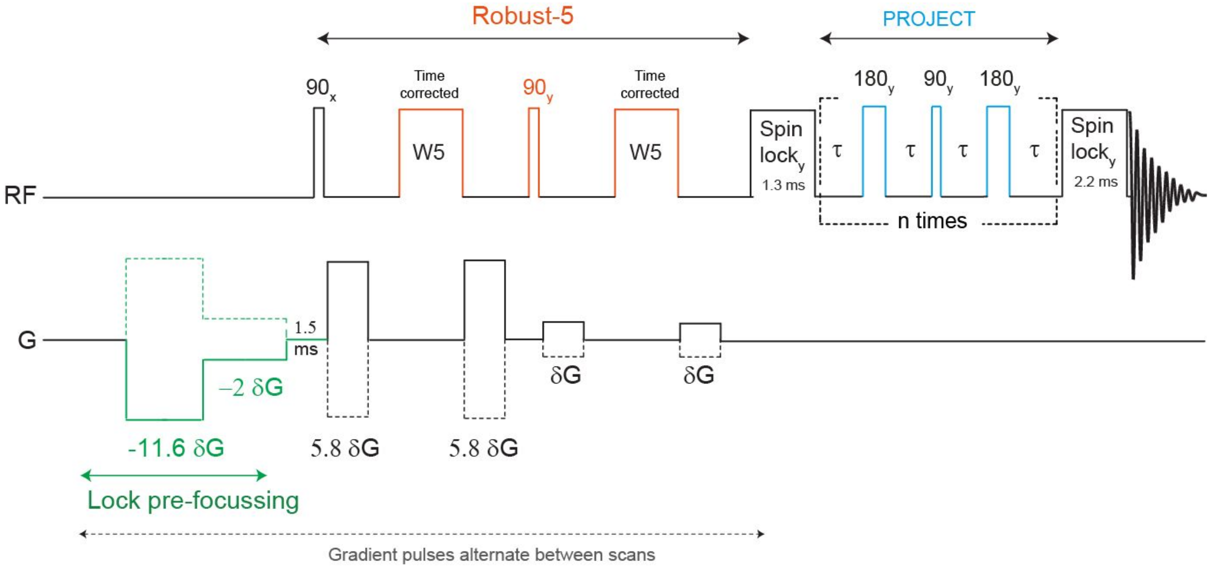

Figure S6. The WASTED-II pulse sequence used to produce Figure 1b. DOI: 10.1039/c9an01005j. WASTED-II is a combination of the PROJECT-T_2-filter and the Robust-5 solvent signal suppression module.

------------------ WASTED-II CODE: START --------remove this line-------------

```
;Email j.a.aguilar@durham.ac.uk for support.
;There is no warranty (implied or explicit) that it is optimal or bug-free.
;Anyone using this code does so at their own risk.

;Reliable, high-quality suppression of NMR signals arising from water and macromolecules:
;application to bio-fluid analysis
;DOI:10.1039/C9AN01005J

;The fist spinlock helps keeping the water signals attenuated.
;The second spinlock eliminates dispersive components.
;Juan A. Aguilar. j.a.aguilar@durham.ac.uk
;1st draft: 20/12/2012 Durham (UK).
;Avance-version (13/05/16)

;Water suppression by Robust-5
;Broad signal suppression by PROJECT

;Robust NMR water signal suppression for demanding analytical applications
;Juan A. Aguilar and Simon. J. Kenwright
;Analyst, 141, pp236-242 (2016)
;doi: 10.1039/C5AN02121A

;Spin echo NMR spectra without J modulation
;Juan A. Aguilar, Mathias Nilsson, Geoffrey Bodenhausen and Gareth A. Morris
;Chem. Commun., 2012,48, 811-813
;doi: 10.1039/C1CC16699A

;$CLASS=HighRes
;$DIM=1D
;$TYPE=
;$SUBTYPE=
;$COMMENT=

#include <Avance.incl>
#include <Grad.incl>
#include <Delay.incl>

"p20=p16*2"
"d19= (1/(2*cnst10))"
"p27=p1"

"d3=l4*d2" ; Total T2 time

"d20=(d19*2)- (2*(p27*0.087 + p27*0.206)/3.1416)"
"d21=(d19*2)- (2*(p27*0.413 + p27*0.206)/3.1416)"
"d22=(d19*2)- (2*(p27*0.413 + p27*0.778)/3.1416)"
"d23=(d19*2)- (2*(p27*0.778 + p27*1.491)/3.1416)"
"d24=(d19*2)- (2*(p27*1.491 + p27*1.491)/3.1416)"
"d25=(d19*2)- (2*(p27*0.778 + p27*1.491)/3.1416)"
```

```
"d26=(d19*2)- (2*(p27*0.413 + p27*0.778)/3.1416)"
"d27=(d19*2)- (2*(p27*0.413 + p27*0.206)/3.1416)"
"d28=(d19*2)- (2*(p27*0.087 + p27*0.206)/3.1416)"
"acqt0=0u"

1 ze
2 30m
  d1

/******************** Robust-5: START ********************/

  50u UNBLKGRAD
  p20:gp3*EA
  d16
  d16
  p20:gp4*EA
  d16
  d16
  10u

  p1 ph1

  4u
  p16:gp1*EA
  d16
  p27*0.087 ph3
  d20
  p27*0.206 ph3
  d21
  p27*0.413 ph3
  d22
  p27*0.778 ph3
  d23
  p27*1.491 ph3
  d24
  p27*1.491 ph4
  d25
  p27*0.778 ph4
  d26
  p27*0.413 ph4
  d27
  p27*0.206 ph4
  d28
  p27*0.087 ph4
  p16:gp1*EA
  d16
  4u

  p1 ph2        /* Perfect echo */

  4u
```

```
  p16:gp2*EA
  d16
  p27*0.087 ph5
  d20
  p27*0.206 ph5
  d21
  p27*0.413 ph5
  d22
  p27*0.778 ph5
  d23
  p27*1.491 ph5
  d24
  p27*1.491 ph6
  d25
  p27*0.778 ph6
  d26
  p27*0.413 ph6
  d27
  p27*0.206 ph6
  d28
  p27*0.087 ph6
  p16:gp2*EA
  d16 igrad EA
  4u BLKGRAD

/******************** Robust-5: END ********************/

p1*65 ph8    /* Spin lock, important to keep the water well attenuated */

/******************** PROJECT: START ********************/
3 d2*0.25

  p1*2 ph7
  d2*0.25

  p1 ph8          /* Perfect echo */
  d2*0.25
  p1*2 ph7
  d2*0.25

  lo to 3 times l4

/******************** PROJECT: END ********************/

p1*100 ph8    /* Spin lock, important to keep the water well attenuated */
go=2 ph31
  30m mc #0 to 2 F0(zd)
exit
ph1=0 2
ph2=1
ph3=0 0 0 0 0 0 0 0 1 1 1 1 1 1 1 1 2 2 2 2 2 2 2 2 3 3 3 3 3 3 3 3
```

```
ph4=2 2 2 2 2 2 2 2 3 3 3 3 3 3 3 3 0 0 0 0 0 0 0 0 1 1 1 1 1 1 1 1
ph5=0 0 1 1 2 2 3 3
ph6=2 2 3 3 0 0 1 1
ph7=0 /* PROJECT 180 */
ph8=1 /* PROJECT 90 */
ph31=0 2 2 0 0 2 2 0 2 0 0 2 2 0 0 2
;pl1 : f1 channel - power level for pulse (default)
;p1 : f1 channel -  90 degree high power pulse
;p16: homospoil/gradient pulse. 1 ms
;d16: delay for homospoil/gradient recovery. 1 ms
;d1 : relaxation delay; 1-5 * T1
;d19: delay for W5 binomial water suppression. Calculated using cnst10
;cnst10: distance to nulls in Hz for W5 binomial water suppression. The further away the
nulls ;the wide the suppression notch will be.
;ns: 32 * n, total number of scans: NS * TD0.  Newer use an odd number of scans or the
residual ;water signal will be huge.
;ds: 4;
;d2 : Cycle time (2 echo times). T2 filter time= d2*l4. d2=20 ms and l4=4 works well.
;l4: loop for T2 filter. Min value= 1 (2 echoes). T2 filter time= d2*l4
; DO NOT SPIN THE SAMPLE
  ;use gradient ratio:    gp 1 :  gp 2 : gp 3 :   gp 4
;                      62 : 10.68 :  -62 : -10.68

;for z-only gradients:
;gpz1: 62.8%
;gpz2: 10.68%
;gpz3: -62.8%
;gpz2: -10.68%

;use gradient files:
;gpnam1: SMSQ10.100
;gpnam2: SMSQ10.100
;gpnam3: SMSQ10.100
;gpnam4: SMSQ10.100
;
```

------------------- WASTED-II CODE: END --------remove this line--------------

------------- PROJECT-SL pulse sequence CODE: START ----------remove this line------

```
;Juan A. Aguilar. 2025. This PROJECT pulse sequence uses a better phase cycle than the
original ;(thanks to G. A. Morris) and a short spinlock to remove dispersive signals created
when users ;violate the PROJECT condition.

;Email j.a.aguilar@durham.ac.uk for support.
;There is no warranty (implied or explicit) that it is optimal or bug-free.
;Anyone using this code does so at their own risk.


; Juan Aguilar. Mathias Nilsson. Gareth Morris. Manchester. 2011.
; Using the Manchester pulse sequence' phase cycle.
; avance-version
; 1D sequence

#include <Avance.incl>
#include <Grad.incl>
#include <Delay.incl>

"p2=p1*2"
"p3=p1"

1 ze
2 30m
d1 pl1:f1

p1 ph1

; PERFECT ECHO

        ; SPIN ECHO
        3 d20
                p2 ph2
          d20
        ; SPIN ECHO

p3 ph3 /* 90d. Refocusing */

        ; SPIN ECHO
        d20
                p2 ph2
        d20
        ; SPIN ECHO

; PERFECT ECHO

lo to 3 times l4

  4u pl10:f1
  p17 ph3;         short spin-lock
```

```
go=2 ph31
30m mc #0 to 2 F0(zd)
exit

ph1=0 2 0 2 1 3 1 3 2 0 2 0 3 1 3 1
ph2=1 1 1 1 0 0 0 0 3 3 3 3 2 2 2 2
ph3=1 1 3 3 0 0 2 2 3 3 1 1 2 2 0 0
ph31=0 2 0 2 1 3 1 3 2 0 2 0 3 1 3 1

;pl1 : f1 channel - power level for pulse (default)
;p1 : f1 channel - 90 degree excitation pulse
;p2 : f1 channel - 180 degree refocusing pulse
;p3 : f1 channel - 90 degree refocusing pulse
;d1 : relaxation delay; 1-5 * T1
;d20x2 : echo time (d2= 5 ms).
;d20x4: perfect echo time (calculated).
;T2-filter duration: d20 x 4 x l4
;ns: multiples of 4
```

------------- PROJECT-SL pulse sequence CODE: END ----------remove this line------

---------------- T_2-filtered PSYCHE-TSE CODE: START. Remove this line -------------

;Pulse sequence to produce T_2-filtered pure shift data (T2-filtered PSYCHE-TSE) for Bruker
;spectrometers
;JAA, 2025-04-08.

;There is no warranty (implied or explicit) that it is optimal or bug-free.
;Anyone using this code does so at their own risk.

;Process with proc_reset
;
;(1) Foroozandeh, M.; Adams, R. W.; Meharry, N. J.; Jeannerat, D.; Nilsson, M.; Morris, G. A. ;Angew. Chem. Int. Ed. 2014, 53, 6990.
;(2) Foroozandeh, M.; Adams, R. W.; Nilsson, M.; Morris, G. A. J. Am. Chem. Soc. 2014, 136, ;11867.
;(3) Foroozandeh, M.; Adams, R. W.; Kiraly, P.; Nilsson, M.; Morris, G. A. Chem. Commun., 2015

```
;$CLASS=HighRes
;$DIM=2D
;$TYPE=
;$SUBTYPE=
;$COMMENT=

#include <Avance.incl>
#include <Delay.incl>
#include <Grad.incl>

define delay BETA
define delay SWi
define pulse P_in
define delay tauA
define delay tauB


"in0=dw*l31"
"d0=3u"

"tauA=in0/2"
"tauB=dw*2*cnst4"

"DELTA2=dw*l31*2"
"DELTA2=DELTA2"
"DELTA3=p16+d16+d0*2-p1*2/PI+de+dw*2*l30+4u"

"SWi=1000000/(l31*2*dw)"
"SWi=SWi"

"TAU=dw*l31/2"
"l29=2*l31*td1"
```

```
"cnst62=(cnst61/360)*sqrt((2*cnst60)/(p49/2000000))"
"P_in = 1000000.0 / (cnst62*4)"
"BETA = cnst62 / (2.27 * 2) "
"cnst63= (P_in/p1) * (P_in/p1)"
"spw37=plw1/cnst63"

"p31=1000000.0/(cnst51*4)"
"cnst32= (p31/p1) * (p31/p1)"
"spw53=plw1/cnst32"
"spw52=spw53"
"p52=cnst52"
"p53=cnst52"

1 ze
2 10m

d1 pl1:f1

3 p1 ph1
  tauA
  50u UNBLKGRAD
  p16:gp1
  d16
  10u pl0:f1
  d16
  ( center (p52:sp52 ph2):f1 (p52:gp11) )
  d16
  60u
  p16:gp1
  d16
  tauA
  d0

d3*0.5; First part of the relaxation filter

  p16:gp2
  d16
  10u
  d16
  ( center (p49:sp37 ph3):f1 (p49:gp10) )
  d16
  10u
  p16:gp2
  d16

d3*0.5            ; Second part of the relaxation filter

  tauB
  p16:gp3
  d16
```

```
  60u
  d16
  ( center (p53:sp53 ph4):f1 (p53:gp12) )
  d16
  10u pl1:f1
  p16:gp3
  d16
  d0
  50u BLKGRAD
  go=2 ph31
  10m mc #0 to 2 F1QF(caldel(d0, +in0))

exit

ph1= 0 2 0 2 0 2 0 2            ; Hard_90
ph2= 0 0 0 0 1 1 1 1            ; sweep-180
ph3= 0 0 1 1 0 0 1 1            ; beta
ph4= 0 0 0 0 0 0 0 0            ; sweep-180
ph29=0
ph31=0 2 2 0 2 0 0 2            ; Receiver

;pl0 : zero power
;pl1 : high power
;pl9 : power level for presat
;p1  : 90 degree high power pulse
;p16 : duration of CTP gradients (1m)
;p49 : duration of double-chirp PSYCHE pulse element
;p52 : duration of 1st 180-degree swept-frequency pulse
;p53 : duration of 2nd 180-degree swept-frequency pulse
;d0  : incremented delay
;d1  : relaxation delay
;d3:  Relaxation filter time
;d16 : recovery delay for gradients
;spw37 : RF power of double-chirp PSYCHE pulse element
;spw43 : RF power of 1st 180-degree swept-frequency pulse
;spw42 : RF power of 2nd 180-degree swept-frequency pulse
;spnam37: file name for PSYCHE pulse element
;spnam52: file name for 1st 180-degree swept-frequency pulse
;spnam53: file name for 2nd 180-degree swept-frequency pulse
;gpz1: CTP gradient (35%)
;gpz2: CTP gradient (49%)
;gpz3: CTP gradient (77%)
;gpz10: weak gradient during PSYCHE element (1-3%)
;gpz11: weak gradient during 1st 180-degree chirp (1-3%)
;gpz12: weak gradient during 2nd 180-degree chirp (1-3%)
;gpnam1: SINE.100
;gpnam2: SINE.100
;gpnam3: SINE.100
;gpnam10: RECT.1
;gpnam11: RECT.1
;gpnam12: RECT.1
```

```
;cnst51: RF amplitude for 180-degree chirp pulses (Hz)
;cnst52: duration of TSE pulses in us
;cnst60: sweep width of single chirp element in Crp_psyche.20   [10000 Hz]
;cnst61: desired flip angle (ca. 20 degree)
;cnst62: gammaB1/2PI value for p49:sp37 (in Hz)
;cnst63: scaling factor for power (sp37)
;l29: total number of points in reconstructed FID
;l30: number of complex points at the beginning not to be included
;       in reconstruction
;l31: number of complex points along the acquisition dimension per block
;       block length (DELTA2) about 8 to 10ms
;BETA: flip angle (ca. 20 degree)
;SWi: sweep width in the pseudo dimension
;       should be 2 to 3 times the width of the widest multiplett
;in0: = dw*l31
;td1 : number of t1 increments
;MC2 : QF
```

----------------- T_2-filtered PSYCHE-TSE CODE: END. Remove this line -------------

---------------- Macro to process the T_2-filtered PSYCHE-TSE: START ----

```
/**********************************************-*-C++-*-********/
/*  proc_1d_reset                                   */
/****************************************************************/
/*  Short Description :                                 */
/*  Processes and plots 1D spectra. Uses 'autoplot' for        */
/*  plotting.                                   */
/****************************************************************/
/*  Keywords :                                  */
/*  1D, autoplot, TopSpin PLOT Editor                    */
/****************************************************************/
/*  Description/Usage :                                  */
/*  Basic processing AU program for 1D spectra, using proc_reset
/* to generate the 1D FID from a pseudo-2D starting dataset */
/*  plotting       */
/*  the acquisition range with TopSpin PLOT Editor.           */
/*  Performs ef, apk, sref, abs and autoplot.                 */
/*  If you want to use your individual TopSpin PLOT Editor      */
/*  layout, you can define it in XWIN-NMR with the command      */
/*  'layout'. If there is none, the default layout             */
/*  '1D_X+int.xwp' is used.                           */
/*  Peak Picking will be done in the acquisition range         */
/****************************************************************/
/*  Author(s) :                                   */
/*  Name        : Peter Dvortsak,Sven Cunskis, Sven Augner      */
/*  Organisation: Bruker BioSpin GmbH                    */
/*  Email       : nmr-software-support@bruker.de              */
/****************************************************************/
/*  Name        Date    Modification:                    */
/*  pdv         901010  created                           */
/*  svcu        010102  use XWIN-PLOT for plotting             */
/*  svcu        041001  increase print_to_file options         */
/*  augn        070418  XWP_PP replaced with PP               */
/*  rke         140218  apk/apk0 depending on DIGMOD           */
/*  ptg                                     150728  _reset - copies data into expno+10000   */
/*                                                                    and
reconstruct fid into expno                               */
/****************************************************************/
/*
$Id: proc_1d,v 1.19.6.1 2014/02/19 15:25:59 wem Exp $
*/

/*********************************************/
/* Declare variables */
int     digmod;
float   offset, ph0;
double  sf, sw_p, f1p, f2p, f1porig, f2porig;
char    xwlay[PATH_MAX], portf[PATH_MAX];
```

```
char pulprog[100];
char resetargs[100];
portf[0] = 0;
int nexpno = expno+10000;
int sexpno=expno;
/*********************************************/
/* processing */

WRPA(name, nexpno, procno, disk, user)
DATASET(name, nexpno, procno, disk, user)
DELETEEXPNO(name,sexpno,disk,user)
(void)sprintf(resetargs,"%d 1",sexpno);
XAU("proc_reset",resetargs)

DATASET(name, sexpno, procno, disk, user)
EF
ERRORABORT
FETCHPARS("DIGMOD", &digmod)
if (digmod == 3)
   APK0
else
   APK
;    SREF
ABS
/*********************************************/
/* If no TopSpin PLOT Editor layout is defined  */
/* the default layout will be used  */
FETCHPAR("LAYOUT",xwlay)
if (xwlay[0] == 0)
   STOREPAR("LAYOUT","+/1D_X+int.xwp")

/*********************************************/
/* store the current plotregion */
FETCHPAR("F1P",&f1porig)
FETCHPAR("F2P",&f2porig)

/*********************************************/
/* define the plotregion as big as */
/* the complete acquisition region */
FETCHPARS("OFFSET",&offset)
FETCHPARS("SW_p",&sw_p)
FETCHPARS("SF",&sf)
f1p = offset;
f2p = f1p - sw_p / sf;
STOREPAR("F1P",f1p)
STOREPAR("F2P",f2p)

/*********************************************/
/* create the pick picking listing for TopSpin PLOT Eitor */
PP
```

```
/*********************************************/
/* restore the original plotregion */
STOREPAR("F1P",f1porig)
STOREPAR("F2P",f2porig)

AUTOPLOT

/*********************************************/
/* Adds include file 'printtofile' that manage */
/* the print to file information    */

#include <inc/printtofile>
QUIT
```
---------------- Macro to process the T_2-filtered PSYCHE-TSE: END, remove this line ----

---------------- T_2-filtered J-Resolved PSYCHE CODE: START ----------remove this line------ -------

```
;There is no warranty (implied or explicit) that it is optimal or bug-free.
;Anyone using this code does so at their own risk.

;J-resolved PSYCHE 2D with T2-filtration.
;JAA. 30-06-2024. Introducing d3 around the PSYCHE pulse to produce T2 attenuation.

;$CLASS=HighRes
;$DIM=2D
;$TYPE=
;$SUBTYPE=
;$COMMENT=


#include <Avance.incl>
#include <Delay.incl>
#include <Grad.incl>

"d0=0u"
"in0=inf1/2"
"d12=20u"
"l0=1"

;calculation of psyche pulse power from desired flip angle
"cnst50=(cnst20/360)*sqrt((2*cnst21)/(p49/2000000))"
"p30=1000000.0/(cnst50*4)"
"cnst31= (p30/p1) * (p30/p1)"
"spw37=plw1/cnst31"

"p31=1000000.0/(cnst51*4)"
"cnst32= (p31/p1) * (p31/p1)"
"spw63=plw1/cnst32"
"spw62=spw63"


1 ze
2 d1
3 d12 pl1:f1

  p1 ph1

  if "l0 %2 == 1"
    {
    50u UNBLKGRAD
    p16:gp1
    d16
    d16 pl0:f1
    ( center (p62:sp62 ph2):f1 (p62:gp11) )
    d16
```

```
    p16:gp1
    d16
    50u
    50u

d3*0.5                  ; T2-filter x 0.5
    p16:gp2

    d16
    d16

    ( center (p49:sp37 ph3):f1 (p49:gp10) )

    d16
    p16:gp2
    d16

d3*0.5                  ; T2-filter x 0.5

    50u
    d0
    50u
    p16:gp3
    d16
    d16
    ( center (p63:sp63 ph4):f1 (p63:gp12) )
    d16
    p16:gp3
    d16
    50u BLKGRAD
    d0

    }
  else
    {
    d0
    50u UNBLKGRAD
    p16:gp3
    d16
    d16
    ( center (p63:sp63 ph4):f1 (p63:gp12) )
    d16
    p16:gp3
    d16
    50u
    d0
    50u

d3*0.5                  ; T2-filter x 0.5
```

```
    p16:gp2
    d16
    d16

    ( center (p49:sp37 ph3):f1 (p49:gp10) )

    d16
    p16:gp2
    d16

d3*0.5                       ; T2-filter x 0.5


    50u
    50u
    p16:gp1
    d16
    d16
    ( center (p62:sp62 ph2):f1 (p62:gp11) )
    d16
    p16:gp1
    d16
    50u BLKGRAD

    }

  go=2 ph31
  d1 mc #0 to 2 F1EA(calclc(l0, 1), caldel(d0, +in0))
exit


ph1 = 0                       ; Hard_90
ph2 = 0                        ; sweep-180
ph3 = 0 1 2 3                  ; PSYCHE
ph4 = 0                       ; sweep-180
ph31=0 2 0 2                    ; Receiver

;ph1= 0 2 0 2 0 2 0 2
;ph2= 0 0 0 0 0 0 0 0
;ph3= 0 0 0 0 1 1 1 1
;ph4= 0 0 1 1 0 0 1 1
;ph31=0 2 2 0 2 0 0 2

;pl0 : zero power
;pl1 : high power
;p1  : 90 degree high power pulse
;p16 : duration of CTP gradients (1m)
;p49 : duration of double-chirp PSYCHE pulse element
;p62 : duration of 1st 180-degree swept-frequency pulse
;p63 : duration of 2nd 180-degree swept-frequency pulse
;d0  : incremented delay
```

;d1 : relaxation delay
;d3 : T2-filter time (s)
;d16 : recovery delay for gradients
;spw37 : RF power of double-chirp PSYCHE pulse element
;spw43 : RF power of 1st 180-degree swept-frequency pulse
;spw42 : RF power of 2nd 180-degree swept-frequency pulse
;spnam37: file name for PSYCHE pulse element
;spnam62: file name for 1st 180-degree swept-frequency pulse
;spnam63: file name for 2nd 180-degree swept-frequency pulse
;gpz1: CTP gradient (35%)
;gpz2: CTP gradient (49%)
;gpz3: CTP gradient (77%)
;gpz10: weak gradient during PSYCHE element (1-3%)
;gpz11: weak gradient during 1st 180-degree chirp (1-3%)
;gpz12: weak gradient during 2nd 180-degree chirp (1-3%)
;gpnam1: SINE.100
;gpnam2: SINE.100
;gpnam3: SINE.100
;gpnam10: RECT.1
;gpnam11: RECT.1
;gpnam12: RECT.1
;cnst20: desired flip angle for PSYCHE pulse element (degree) (normally 10-25)
;cnst21: bandwidth of each chirp in PSYCHE pulse element (Hz) (normally 10000)
;cnst51: RF amplitude for 180-degree chirp pulses (Hz)
;l0 : loop for N/R cycle
;in0 : 1/(2 * SW) = DW
;nd0 : 2
;td1 : number of t1 increments
;MC2 : EA

;Process the experiment using the xfb;tilt

----------------- T_2-filtered J-Resolved PSYCHE: END -----------------------

The PROJECT-DOSY pulse sequence:

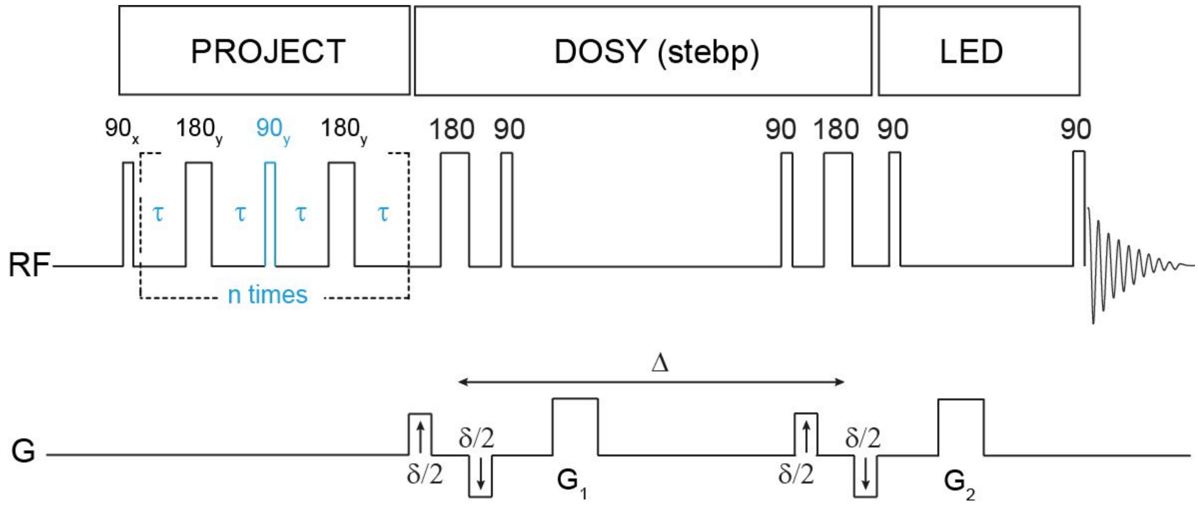

Figure S7. The PROJECT-DOSY pulse sequence used to produce Figure 4.

-------------------- PROJECT-DOSY CODE: START ----------remove this line---------

```
;JAA. PROJECT-DOSY. Constructed using PROJECT and ledbpgp2s
; Contact j.a.aguilar@durham.ac.uk for assistance.

;There is no warranty (implied or explicit) that it is optimal or bug-free.
;Anyone using this code does so at their own risk.


;avance-version (21/09/25)
;2D sequence for diffusion measurement using stimulated
;   echo and LED
;using bipolar gradient pulses for diffusion
;using 2 spoil gradients
;
;D. Wu, A. Chen & C.S. Johnson Jr., ;   J. Magn. Reson. A 115, 260-264 (1995).
;
;$CLASS=HighRes
;$DIM=2D
;$TYPE=
;$SUBTYPE=
;$COMMENT=


#include <Avance.incl>
#include <Grad.incl>
#include <Delay.incl>

define list<gradient> diff=<Difframp>

"p2=p1*2"

"DELTA=d1-30m-4u"
"DELTA1=d20-p1*2-p2-p30*2-d16*2-p19-d16-20u"
"DELTA2=d21-p19-d16-4u"

"acqt0=-p1*2/3.1416"

1 ze
2 30m

#   ifdef FLAG_BLK
  4u LOCKH_OFF
#   else
  4u
#   endif /*FLAG_BLK*/

  DELTA

  p1 ph1                ; FIRST 90
```

```
;;;;;;;;;; PROJECT ;;;;;;;;;

3 d2*0.25
  p2 ph6
  d2*0.25

p1 ph7          ; Perfect echo

  d2*0.25
  p2 ph6
  d2*0.25

  lo to 3 times l4

;;;;;;;;;; PROJECT ;;;;;;;;;

  50u UNBLKGRAD

  p30:gp6*diff
  d16
  p2 ph1
  p30:gp6*-1*diff
  d16
  p1 ph2
  p19:gp7
  d16

#   ifdef FLAG_BLK
  DELTA1 BLKGRAMP
  20u UNBLKGRAMP
#   else
  DELTA1
  20u
#   endif /*FLAG_BLK*/

  p1 ph3
  p30:gp6*diff
  d16
  p2 ph1
  p30:gp6*-1*diff
  d16
  p1 ph4
  p19:gp8
  d16

#   ifdef FLAG_BLK
  DELTA2 BLKGRAMP
  4u
#   else
  DELTA2
  4u BLKGRAD
```

```
#   endif /*FLAG_BLK*/

  p1 ph5
  go=2 ph31
  30m mc #0 to 2 F1QF(calgrad(diff))
#   ifdef FLAG_BLK
  4u BLKGRAD
#   else
  4u
#   endif /*FLAG_BLK*/
exit

ph1= 0
ph2= 0 0 2 2
ph3= 0 0 0 0 2 2 2 2  1 1 1 1 3 3 3 3
ph4= 0 2 0 2 2 0 2 0  1 3 1 3 3 1 3 1
ph5= 0 0 0 0 2 2 2 2  1 1 1 1 3 3 3 3
ph6= 2      ; Perfect echo 180
ph7= 1      ; Perfect echo 90
ph31=0 2 2 0 2 0 0 2  3 1 1 3 1 3 3 1


;pl1 : f1 channel - power level for pulse (default)
;p1  : f1 channel -  90 degree high power pulse
;p2  : f1 channel - 180 degree high power pulse
;p19: gradient pulse 2 (spoil gradient)
;p30: gradient pulse (little DELTA * 0.5)
;d1  : relaxation delay; 1-5 * T1
;d16: delay for gradient recovery
;d20: diffusion time (big DELTA)
;d21: eddy current delay (Te)   [5 ms]
;ns: 8 * n
;ds: 4 * m
;td1: number of experiments
;FnMODE: QF
;       use xf2 and DOSY processing

;T2-filter time= l4 * d2
;l4  ; numer of perfect echoes
;d2 ; perfect echo time (two spin-echoes)

;use gradient ratio:    gp 6 : gp 7   : gp 8
;                       100  : -17.13 : -13.17

;for z-only gradients:
;gpz6: 100%
;gpz7: -17.13% (spoil)
;gpz8: -13.17% (spoil)

;use gradient files:
;gpnam6: SMSQ10.100 or Difftrap
```

;gpnam7: SMSQ10.100
;gpnam8: SMSQ10.100

;preprocessor-flags-start
;zgoptns: option -DFLAG_BLK for alternative gradient amplifier blanking and LOCK_HOLD handling
;   FLAG_BLK: always blank gradient amplifier and lock only active during D1
;preprocessor-flags-end

;use AU-program dosy to calculate gradient ramp-file Difframp

;$Id: ledbpgp2s,v 1.7.8.1 2012/01/31 17:56:33 ber Exp $

-------------------- PROJECT-DOSY pulse sequence: END -----remove this line--------------

The convection test pulse sequence:

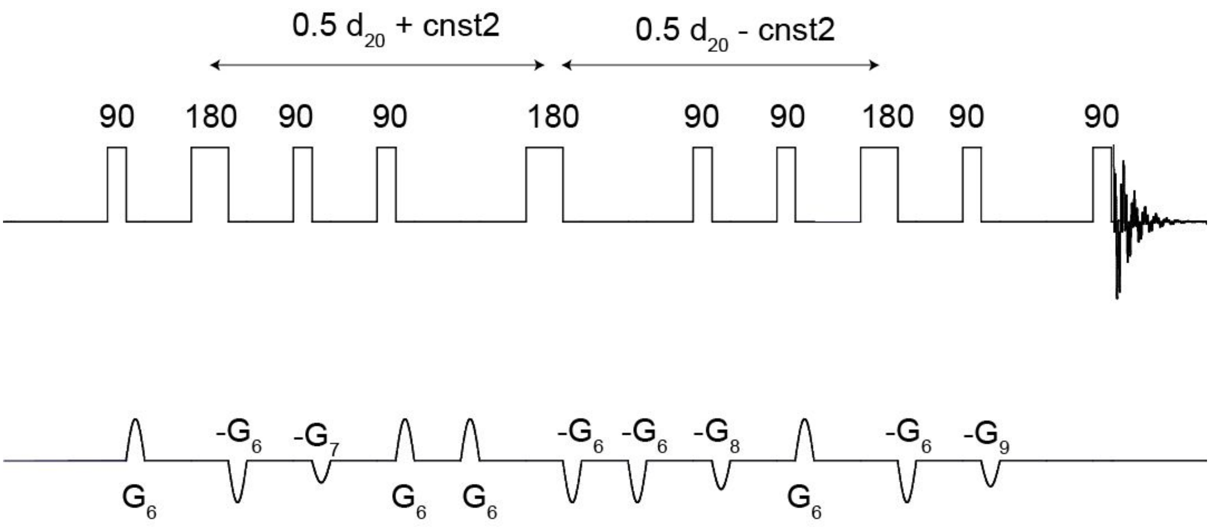

Figure S8. The convection test pulse sequence.

The convection test consists of running a series of 1D convection compensated pulse sequences progressively un-compensating the pulse sequence while maintaining the diffusion time constant (d_{20}). This is done adding time (cnst2) to the first stimulated echo and subtracting it from the second. This is done using the paropt command:

First, define the signal to be monitored by zooming in the signals and typing dpl. Then, typer paropt and answer the following:

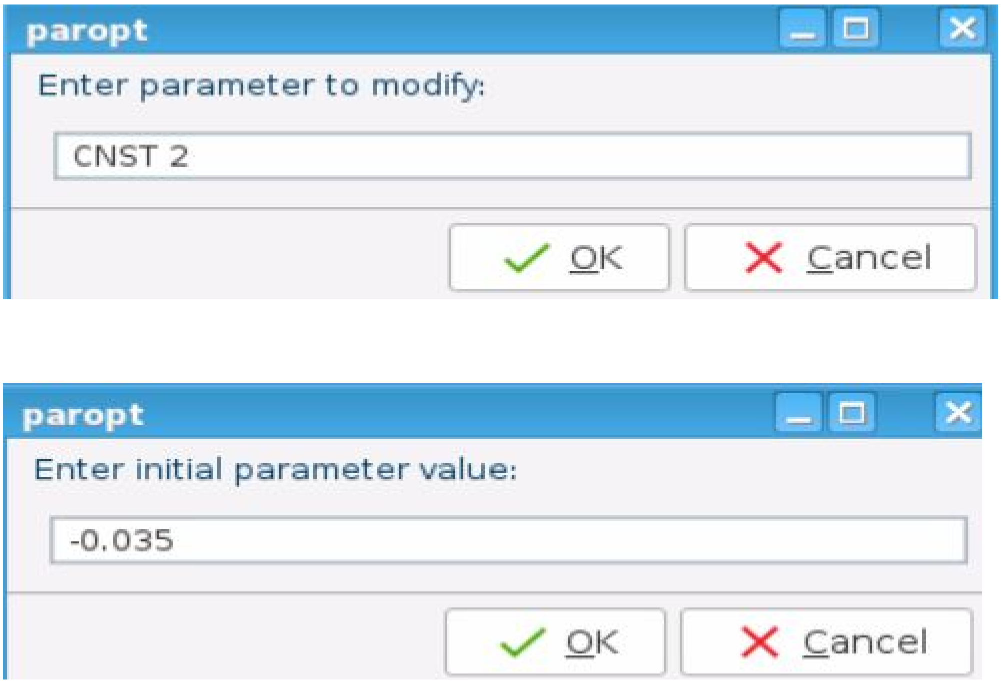

Figure S9. How to run a convection test. See text for details.

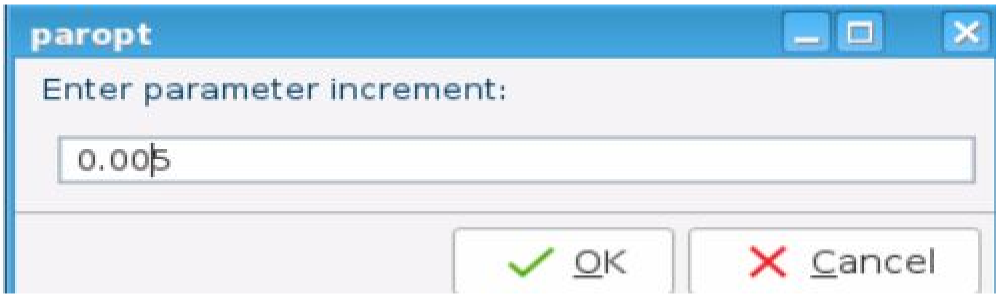

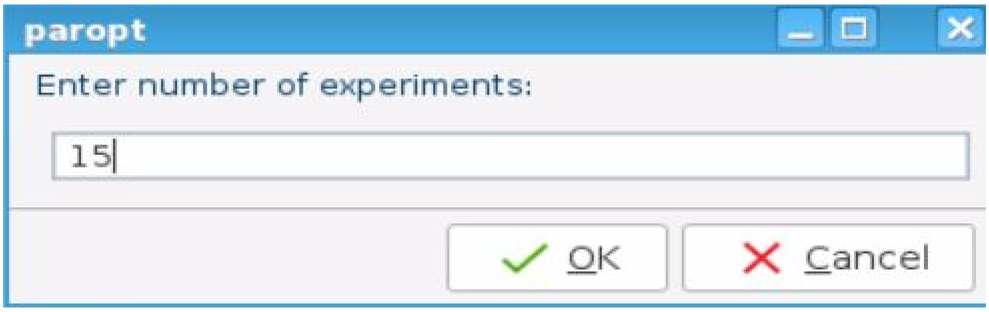

In our case the diffusion time (d_{20}) was 200 ms. The result will appear in processing number 999.

If there is no convection is present, adding some time (cnst2) to the first stimulated echo and subtracting from the second has no effect. One then obtains, as in our case:

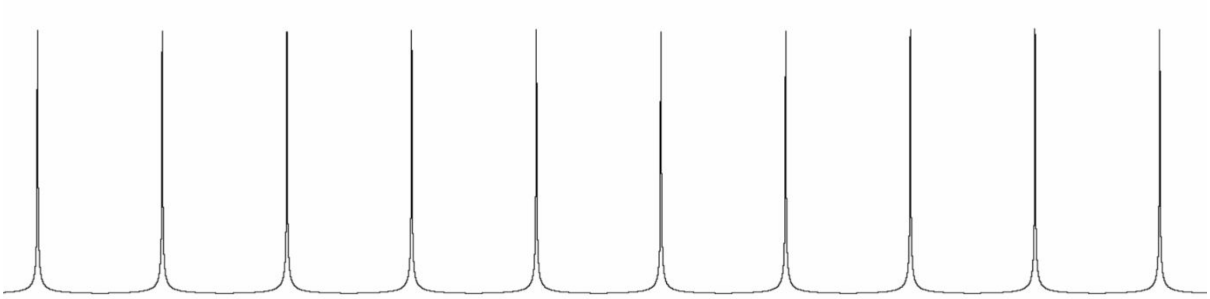

Figure S10. The result of a convection test applied to a D_2O sample at 25 °C. Each signal is the result of incrementing **cnst2** by 5 ms each time. Notice the flat-shaped profile. This shows that there is no convection.

If there is convection, one would obtain a bell-shaped form. For example, a convecting $CDCl_3$ sample produced the following:

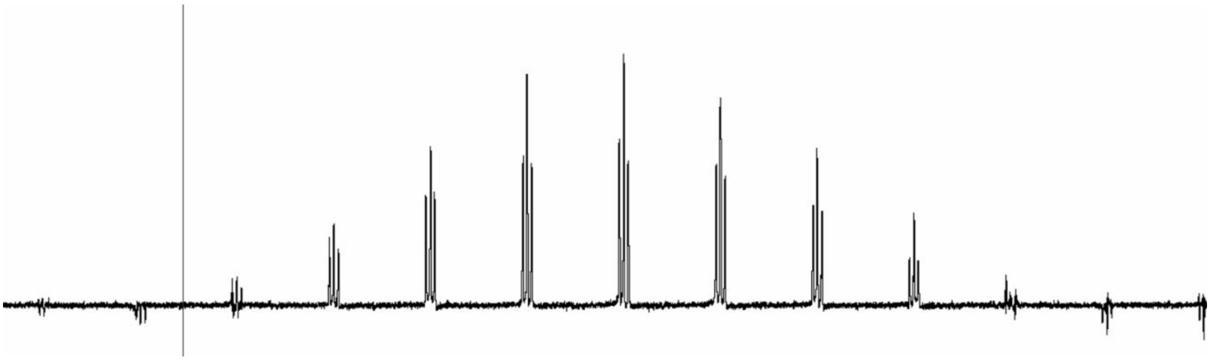

Figure SI- 4. A positive convection test produced using a $CDCl_3$ sample at 25 °C. Notice the bell-shaped profile. Only the maximum intensity is obtained when no extra time is added or subtracted.

------------------- PROJECT-DOSY pulse sequence: START -----remove this line--------------

```
;Convection test. Unbalancing dstebpgp3s1d using cnst2. JAA 16-06-2022
;avance-version (12/01/31)

; Contact j.a.aguilar@durham.ac.uk for assistance.

;There is no warranty (implied or explicit) that it is optimal or bug-free.
;Anyone using this code does so at their own risk.


;$CLASS=HighRes
;$DIM=1D
;$TYPE=
;$SUBTYPE=
;$COMMENT=
;A. Jerschow & N. Mueller, J. Magn. Reson. A 125, 372-375 (1997)


#include <Avance.incl>
#include <Grad.incl>
#include <Delay.incl>


"p2=p1*2"

"DELTA1=(d20-p30*6-d16*6-p19*2-p1*4-p2*2)*0.5"
"DELTA2=d21-p19-4u"
"DELTA3=DELTA1 + cnst2"
"DELTA4=DELTA1 - cnst2"

"acqt0=-p1*2/3.1416"


1 ze
2 d1
  50u UNBLKGRAD
  p1 ph1      ; 1st 90

  p30:gp6
  d16

  p2 ph2      ; 180

  p30:gp6*-1
  d16

  p1 ph3      ; 2nd 90
```

```
  p19:gp7

DELTA3

  p1 ph4        ; 3rd 90


  p30:gp6
  d16

  p30:gp6
  d16

  p2 ph5        ; 180

  p30:gp6*-1
  d16
  p30:gp6*-1
  d16


  p1 ph6           ; 2nd 90

  p19:gp8

DELTA4

  p1 ph3       ; 90

  p30:gp6
  d16

  p2 ph8         ; 180

  p30:gp6*-1
  d16

  p1 ph3        ; 90 LED

  p19:gp9

  DELTA2

  4u BLKGRAD

  p1 ph3           ; 90 LED

  go=2 ph31
  30m mc #0 to 2 F0(zd)
exit
```

```
ph1= 0 0 1 1 2 2 3 3
ph2= 1 2 2 3 3 0 0 1
ph3= 0
ph4= 2 2 3 3
ph5= 3 3 0 0 3 3 0 0  0 0 1 1 0 0 1 1
ph6= 2
ph8= 0 0 0 0 0 0 0 0  0 0 0 0 0 0 0 0
     2 2 2 2 2 2 2 2  2 2 2 2 2 2 2 2
ph31=0 2 0 2 2 0 2 0  2 0 2 0 0 2 0 2


;pl1 : f1 channel - power level for pulse (default)
;p1 : f1 channel -  high power pulse
;p2 : f1 channel - 180 degree high power pulse
;p19: gradient pulse 2 (spoil gradient)
;p30: gradient pulse (little DELTA * 0.5)
;d1 : relaxation delay; 1-5 * T1
;d16: delay for gradient recovery
;d20: diffusion time (big DELTA)
;d21: eddy current delay (Te)                    [5 ms]
;ns: 16 * n, total number of scans: NS * TD0
;ds: 4 * m
;cnst2: unbalancing factor in seconds (try 5 ms).

;use gradient ratio:    gp 6        :  gp 7  : gp 8   : gp 9
;                       26 or more  : -13.17 : -17.13 : -15.37

;for z-only gradients:

;gpz7: -13.17% (spoil)
;gpz8: -17.13% (spoil)
;gpz9: -15.37% (spoil)

;use gradient files:
;gpnam6: SMSQ10.100
;gpnam7: SMSQ10.100
;gpnam8: SMSQ10.100
;gpnam9: SMSQ10.100

;the gradients serve the following purpose:
;   p30
;   p30            first STE dephase bipolar pulse pair
;   p19            spoiler
;   p30
;   p30            first STE rephase bipolar pulse pair
;   p30            and second STE dephase bipolar pulse pair
;   p30
;   p19            spoiler
;   p30
```

;   p30   second STE rephase bipolar pulse pair
;   p19   LED with spoiler

;$Id:$

-------------------- PROJECT-DOSY pulse sequence: END -----remove this line--------------

SECTION III: tutorials

How use apodization to attenuate broad signals in a magnitude mode COSY. Topspin and MestreNova examples.

How use apodization to attenuate broad signals in a HSQC using Topspin or MestreNova.

How to process PROJECT-DOSY in TopSpin.

How use apodization to attenuate broad signals in a magnitude mode COSY

This can be achieved by using window functions to penalize the first points of the fid where broad signals are intense. Without these functions, one obtains a COSY where the broad signals interfere as in here:

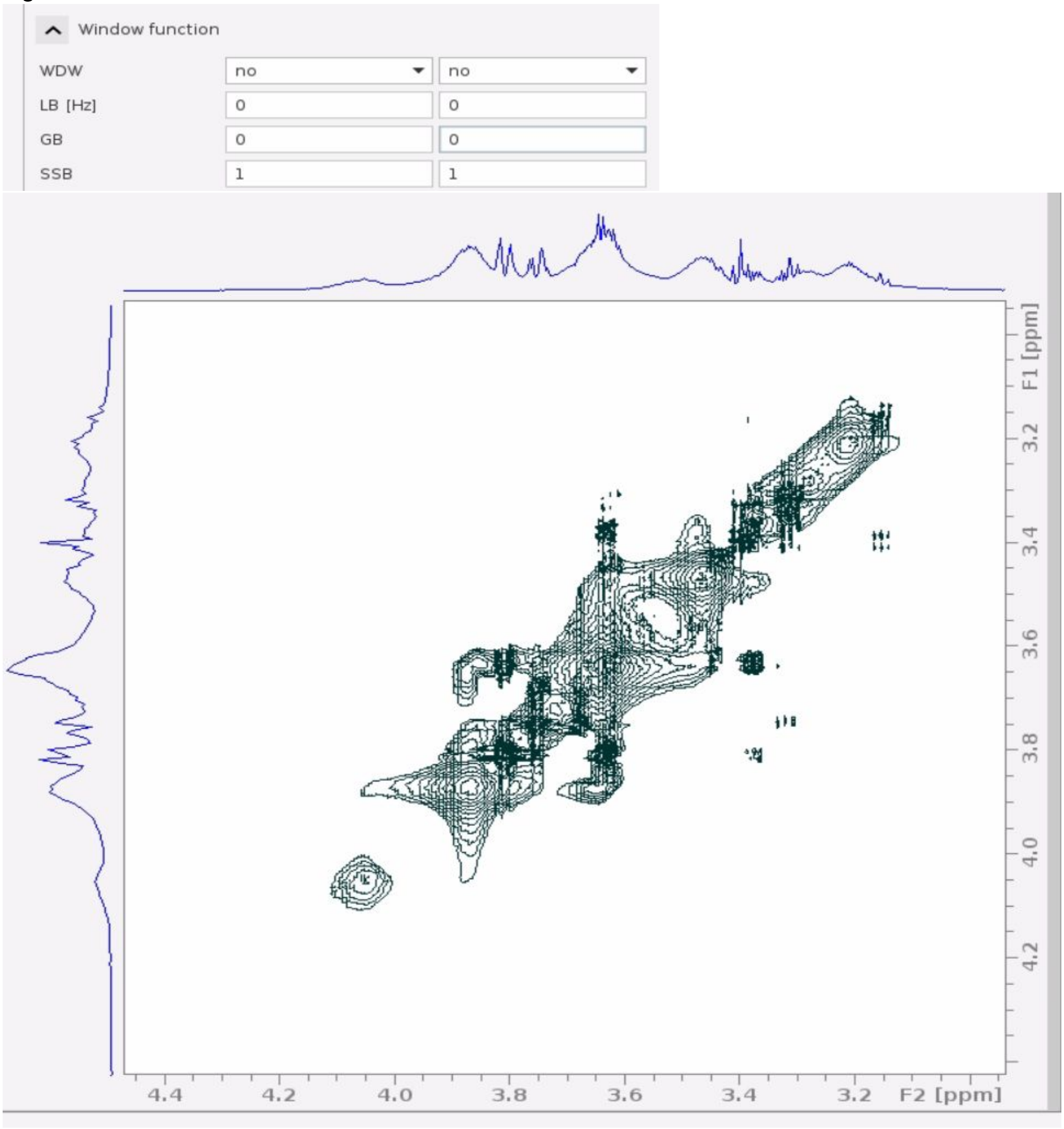

Figure S11. A magnitude-mode COSY processed without apodization. The polymer signals are present (although attenuated). We used TopSpin to process the data.

If a window function is used to penalize the beginning of the fid one obtains a COSY with attenuated or removed broad signals.

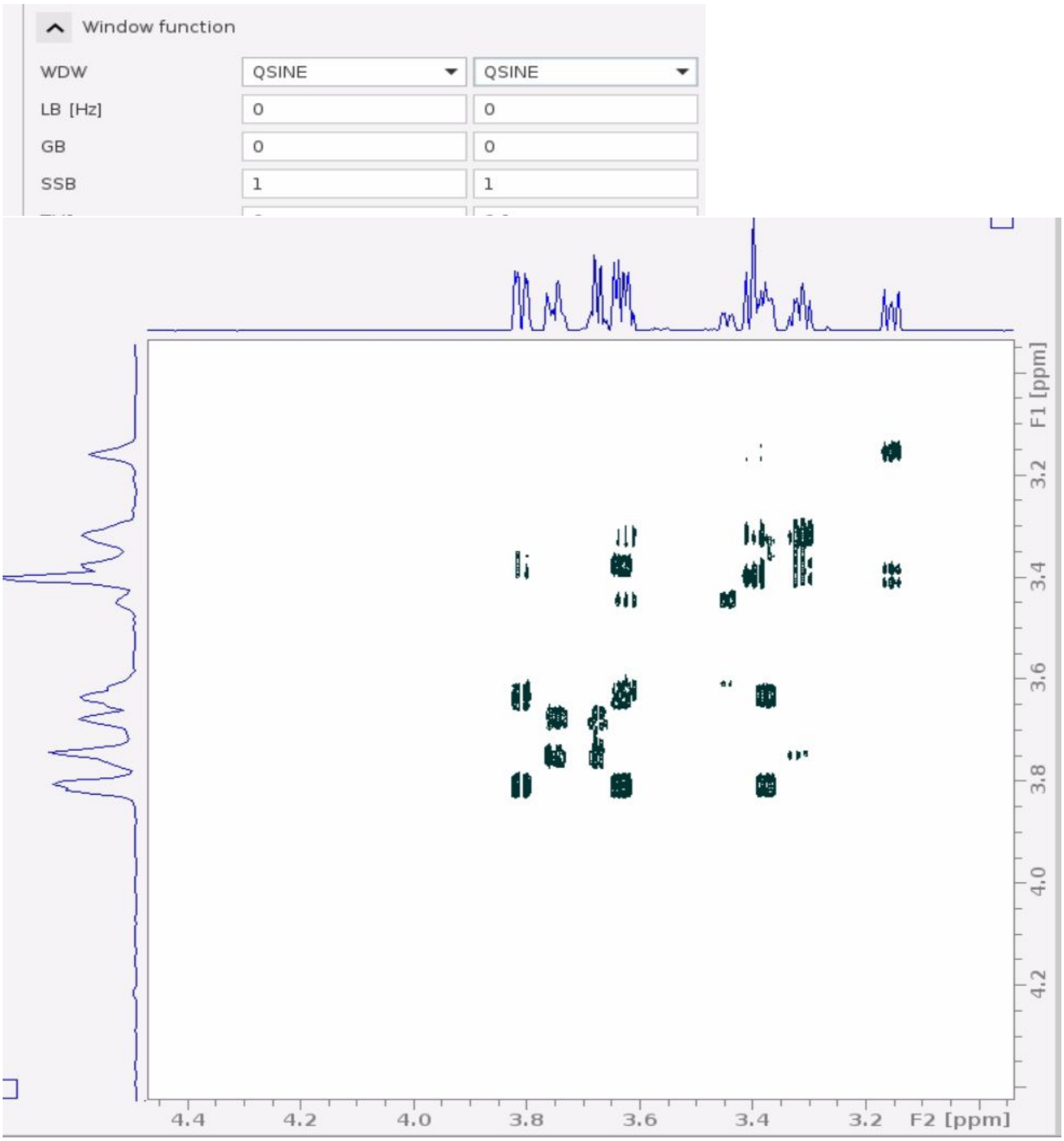

Figure S12. A magnitude-mode COSY processed using sine-squared functions to attenuate broad signals. We used TopSpin to process the data.

Please make sure that the COSY is processed in magnitude mode:

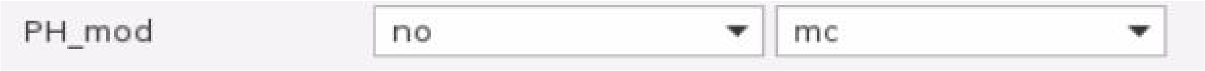

More attenuation can be achieved by moving the size and position of the window function. This is done easily with MestreNova, where one can see the size and position of the function. For example:
With no apodization:

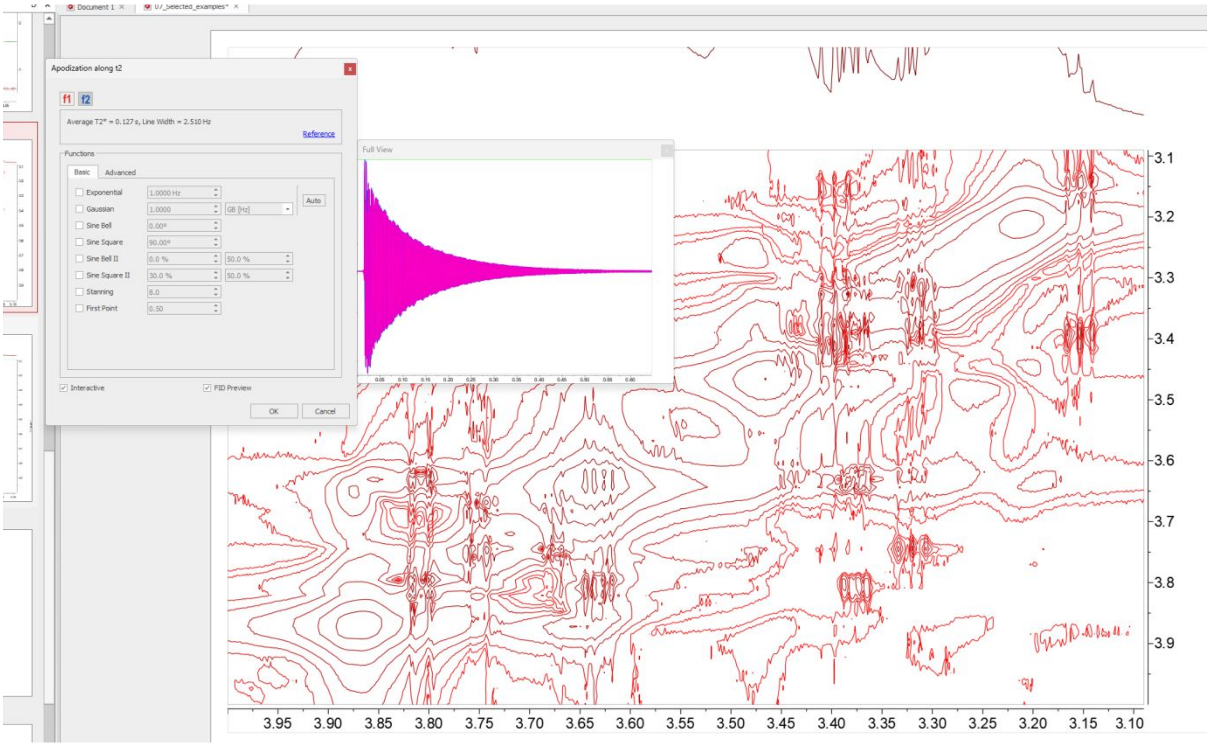

Figure S13. A magnitude-mode COSY processed without apodization. Notice the broad signals. We used MestreNova to process the data.

Now shifting the filter:

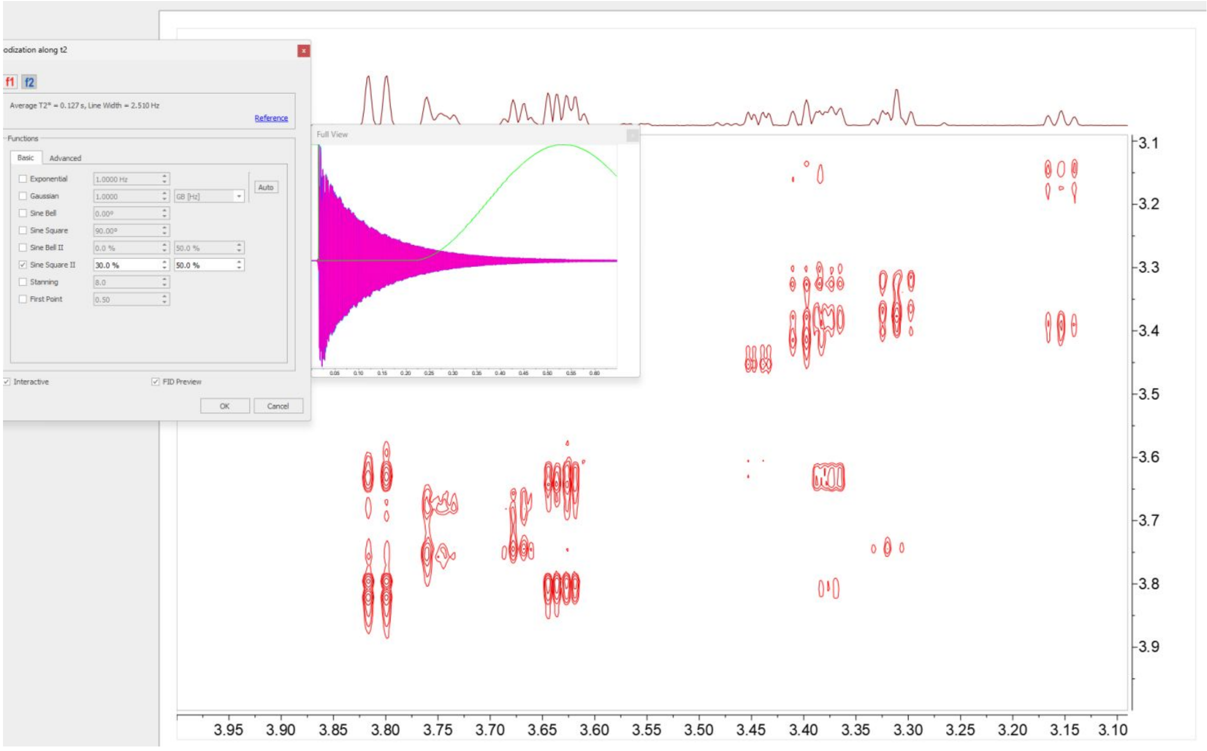

Figure S14. A magnitude-mode COSY processed using sine-squared apodization to attenuate broad signals. We used MestreNova to process the data.

The green line represents the size and position of the filter. Notice how the first points of the fid have been penalized (the fid is multiplied by the filter/apodization function). This will produce a stronger T_2-filtration than the following case, where the first part of the fid has not been as penalized:

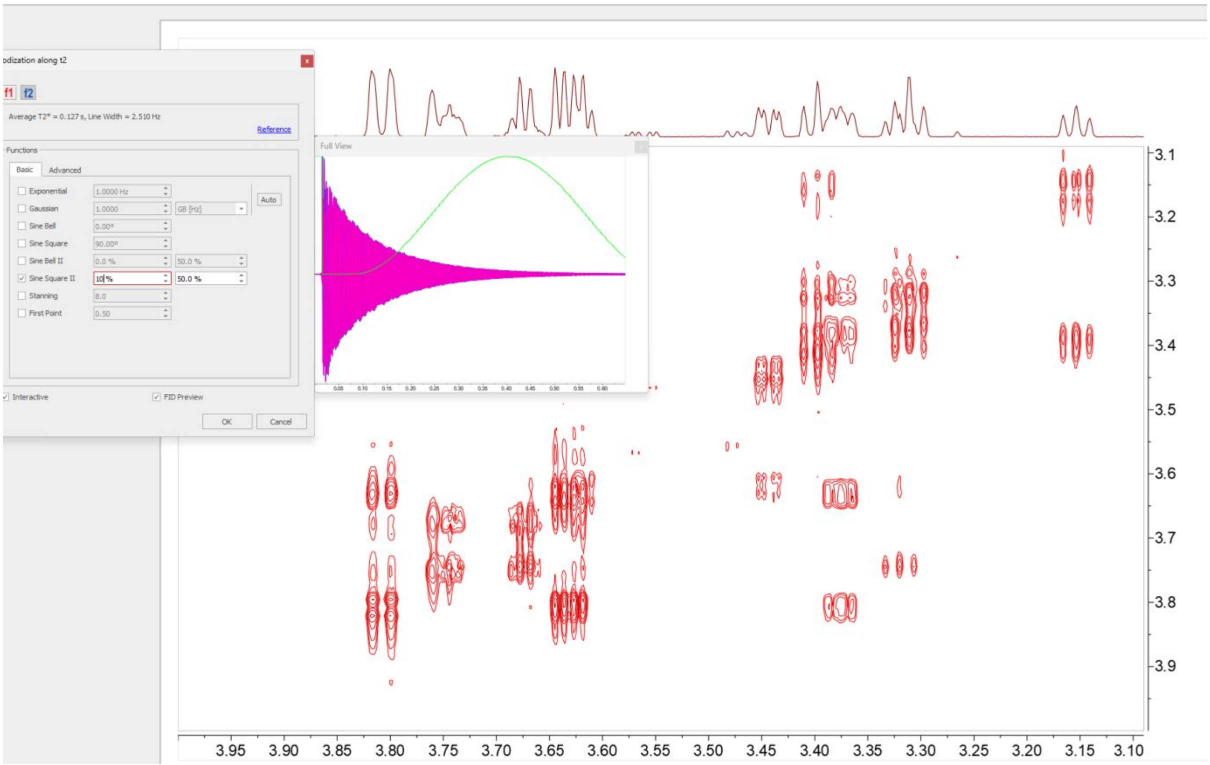

Figure S15. A magnitude-mode COSY processed using sine-squared functions to attenuate broad signals. We used MestreNova to process the data. Notice that we moved the position of the filter towards earlier parts of the fid, so that the degree of T_2-attenuation is lower than in the previous example but that the sensitivity is higher.

How use apodization to attenuate broad signals in a HSQC using Topspin.

Processing the HSQC as usual shows polymer peaks as well as peaks from small molecules:

WDW	QSINE	QSINE
LB [Hz]	0	0
GB	0	0
SSB	2	2
PH_mod	no	pk

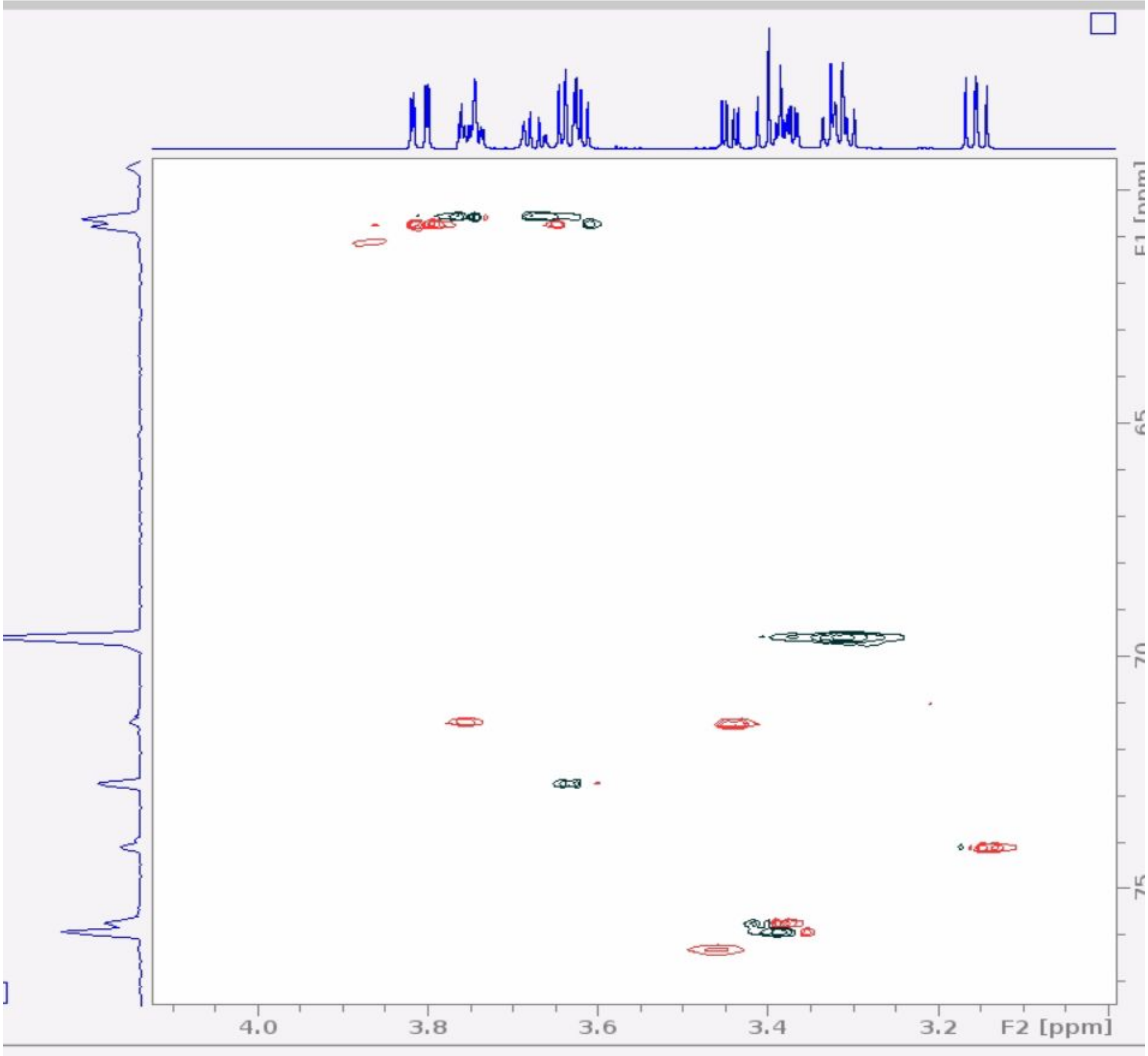

Figure S16. A multiplicity-edited ^{1}H-^{13}C HSQC processed with TopSpin. We did not use any apodization to attenuate broad signals.

To eliminate the signals from the polymer we are going to use the same method we used with the COSY (see above).
First, set the display/processing method to magnitude:

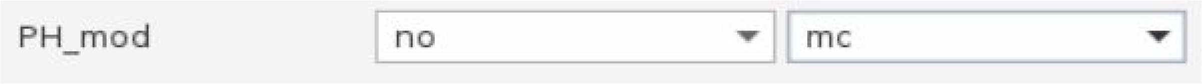

Then set up the sine-square window functions as follows:

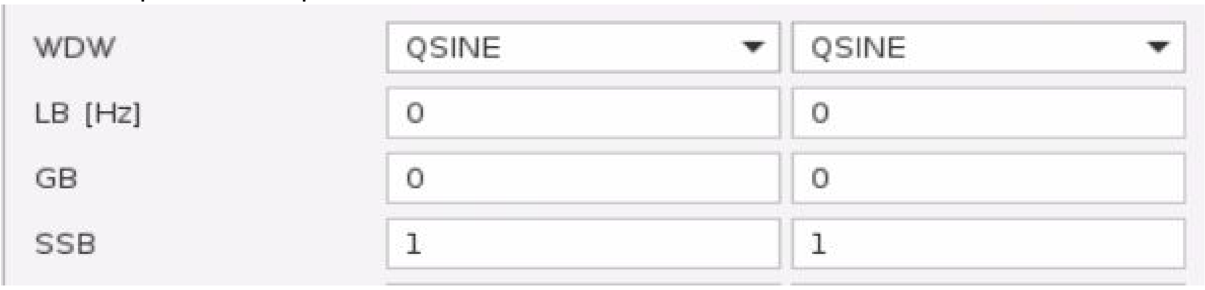

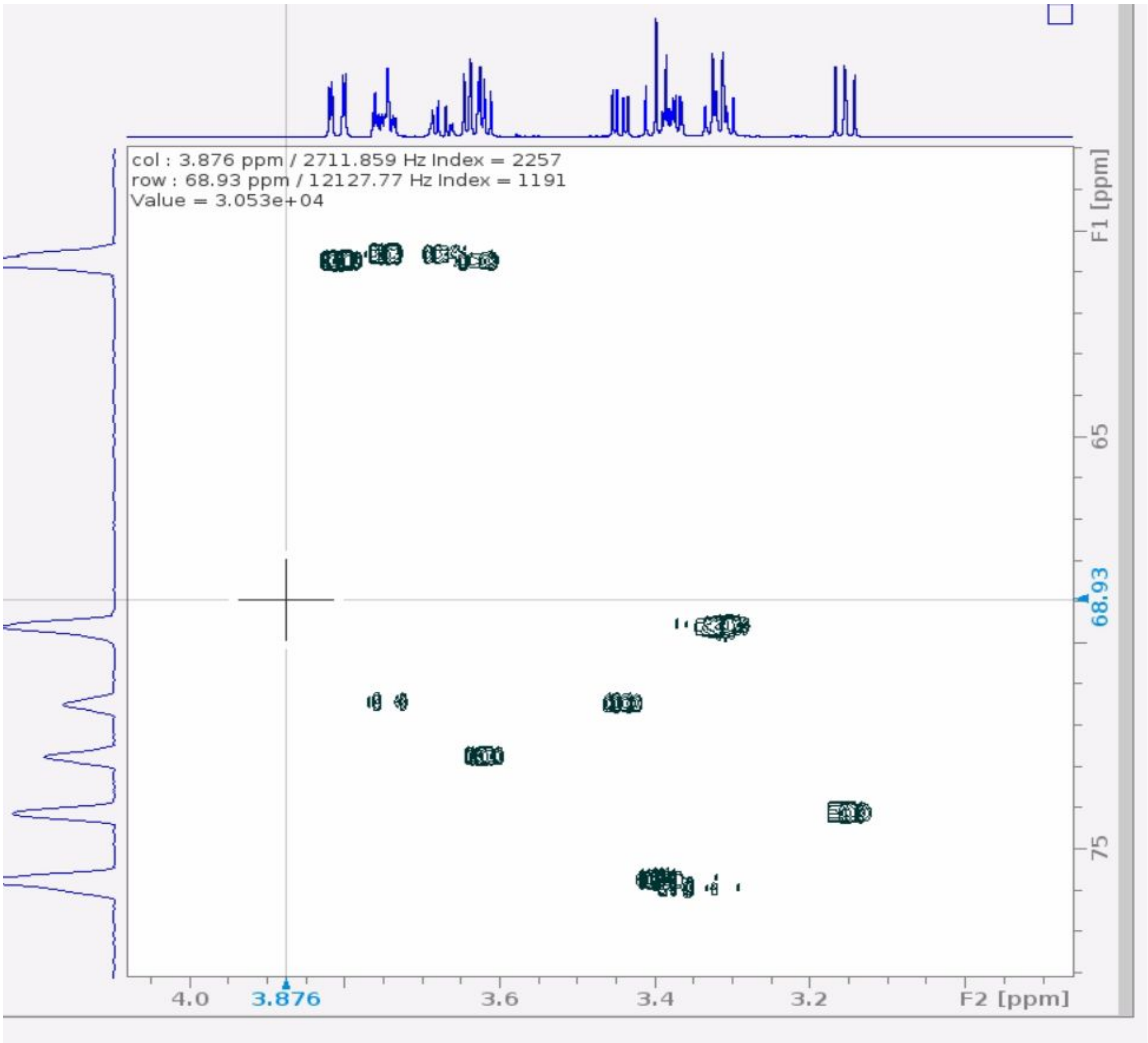

Figure S17. The broad signals can be removed from the ^{1}H-^{13}C HSQC using processing functions (apodization) that penalize the early parts of the fid (as in the COSY case). We used TopSpin to process the data.

How to create T_2-filtration using apodization functions to remove broad signals from the ^{1}H-^{13}C HSQC using MestreNova.

Click on processing template

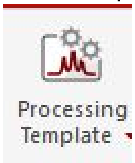

Unclick phase correction in F2:

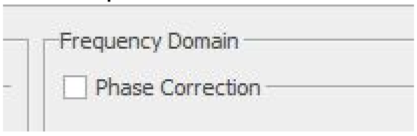

Select magnitude in F1:

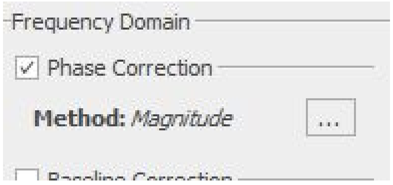

Set up the apodization in F2 function as follows:

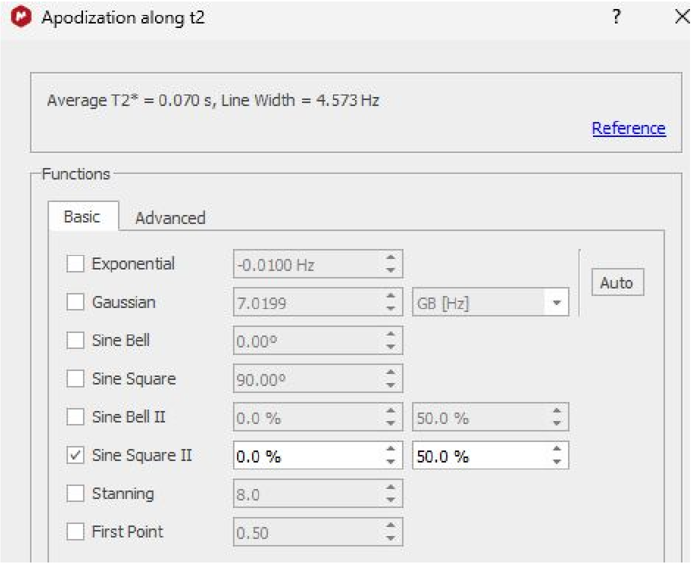

Figure S18. *Windows function used to suppress the broad signals from the ^{1}H-^{13}C HSQC. We used MestreNova to process the spectrum.*

Set up the apodization in F1 function as follows:

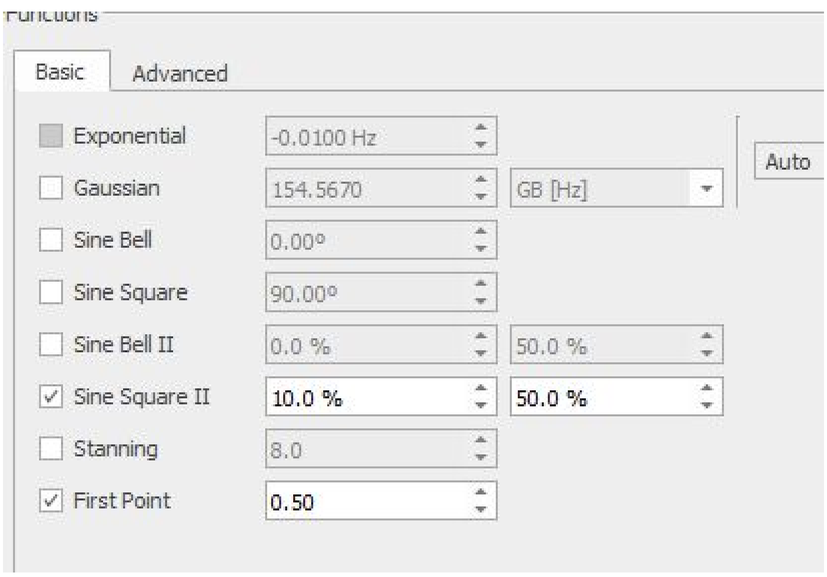

You can change the parameters as in the COSY to change the position and size of the filter to balance the suppression of the broad signal and sensitivity.

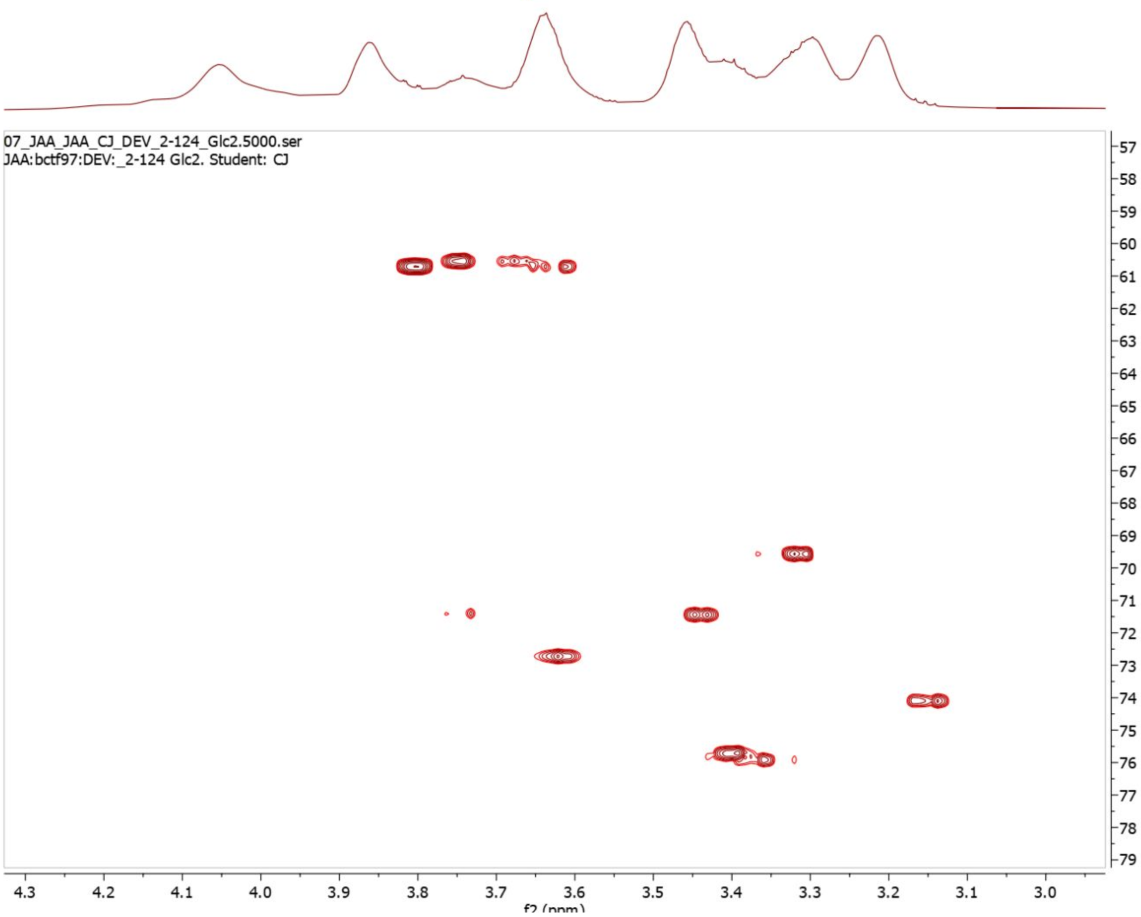

Figure S19. How to suppress broad signals from the previous HSQC using MestreNova.

How to process PROJECT-DOSY with TopSpin

Process the first increment using **efp 1 101 y**

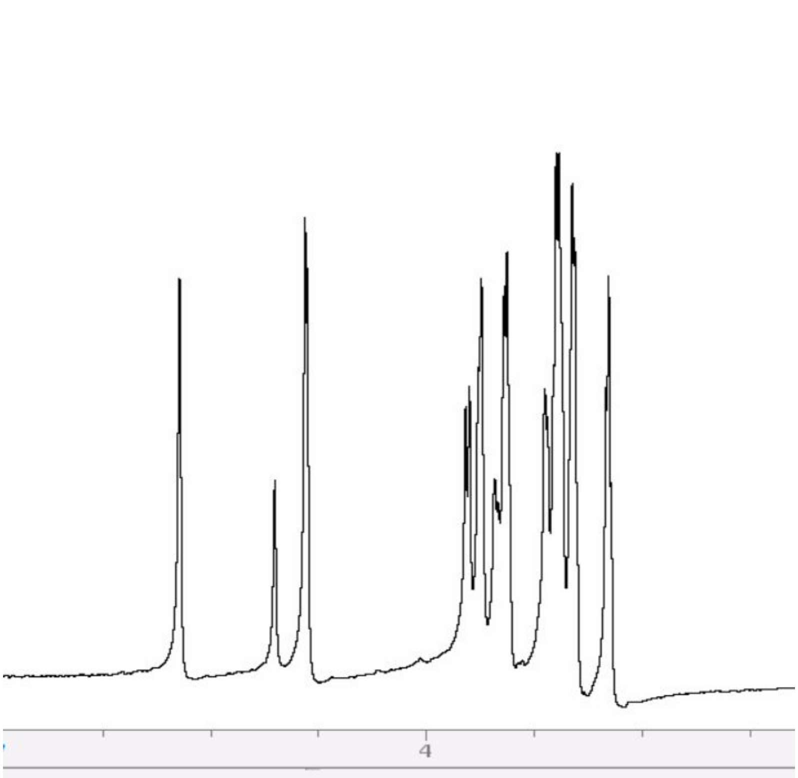

Figure S20. The first increment before correcting the phase.

Phase the increment: **.ph**

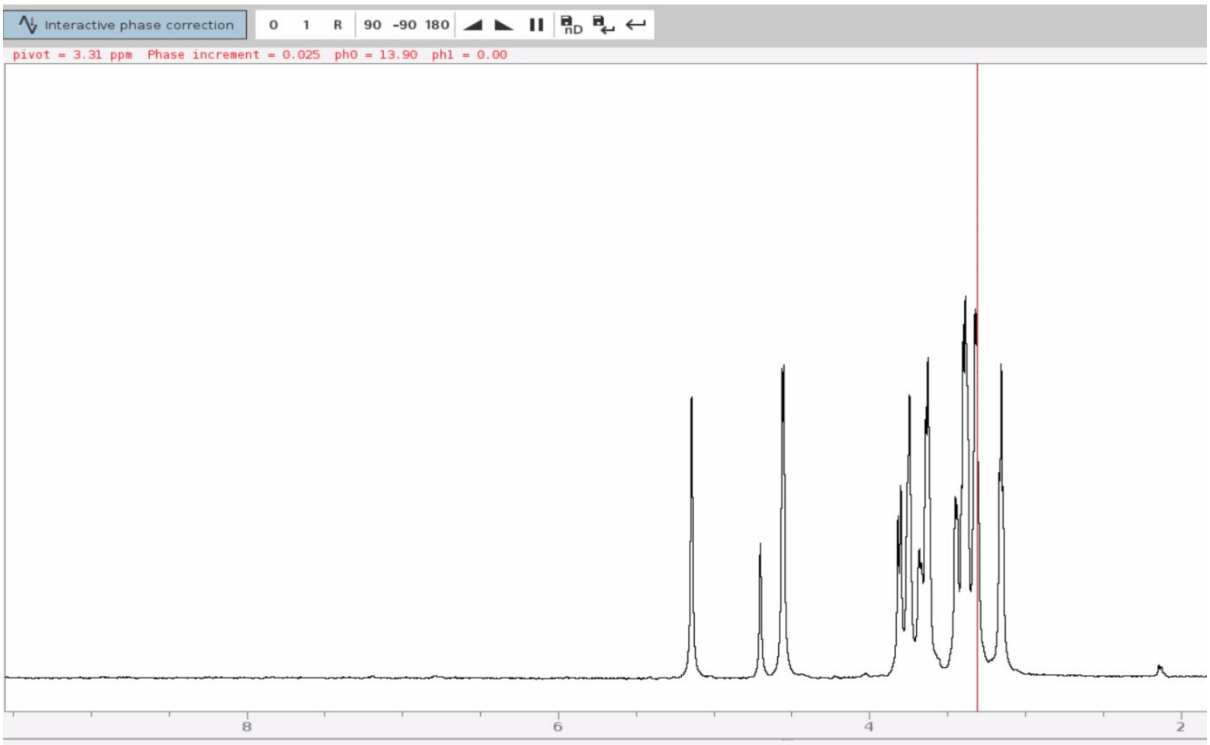

Figure S21.The first increment after correcting the phase.

Save the phase for the 2D dataset: 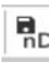

Return to the 2D dataset: **rep 1**

Set the number of points to describe the diffusion dimension to 512 (at least):

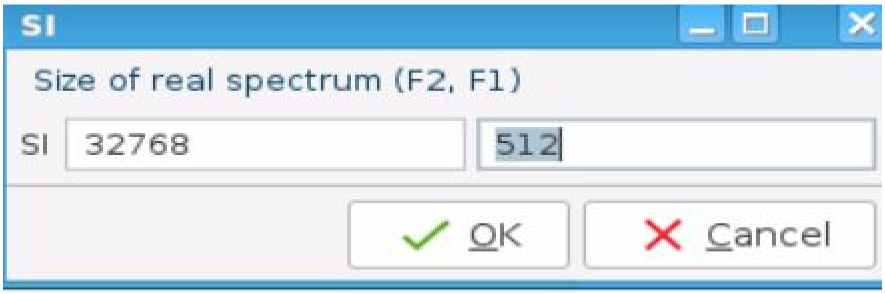

This is not the number of diffusion increments acquired, just the number of points used to describe the diffusion dimension. A small number will make the peaks in the diffusion dimension unnecessarily broad.

Process it using **xf2:**

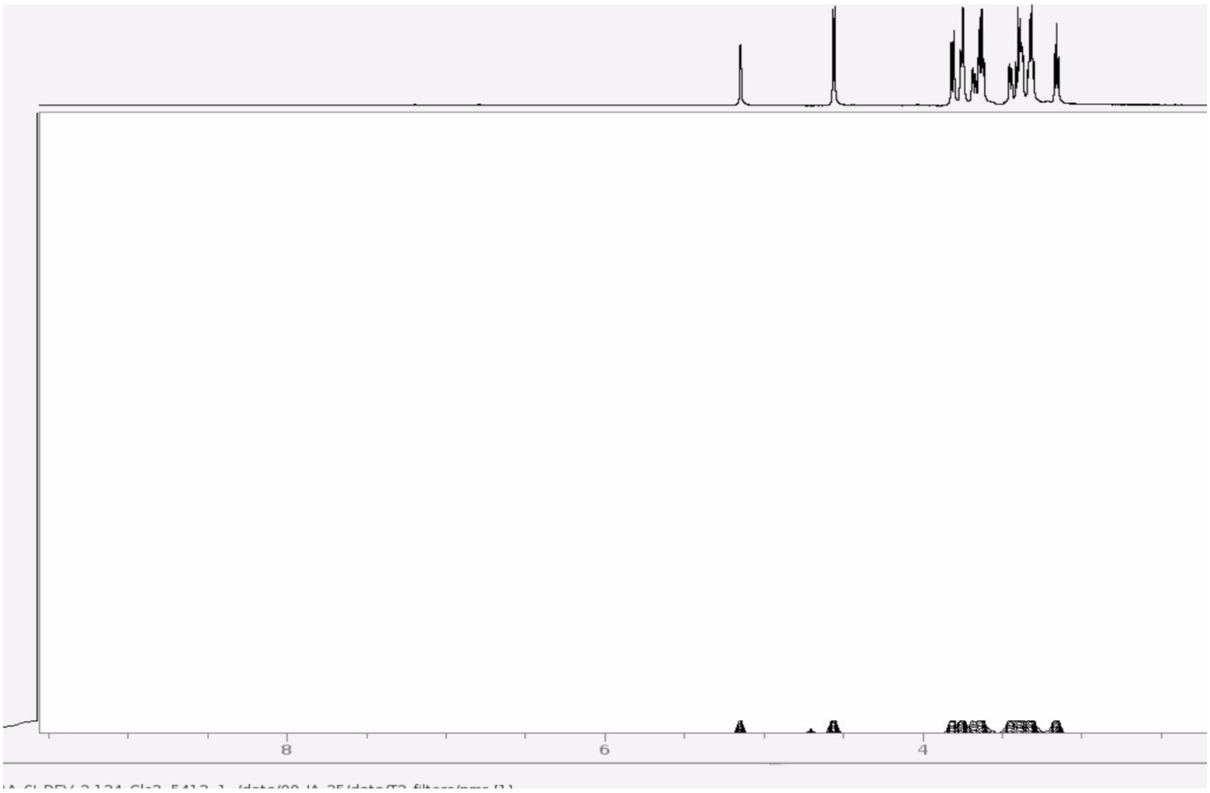

Correct the baseline using **abs2**.

Transfer parameters to the diffusion module using **setdiffparm STEbp**

The **setdiffparm STEbp** tells the DOSY module to use the diffusion equation adequate for a stimulated-echo pulse sequence that used bipolar pulses.

Type **eddosy** to enter the DOSY module:

The important options are:

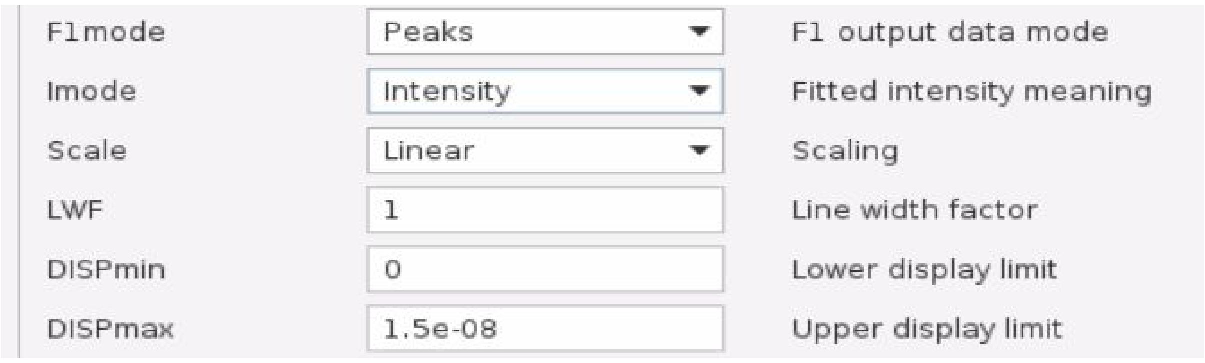

Figure S22. Setting up the DOSY parameters after eddosy.

Use **peaks** and **intensity**. The integral mode is not adequate as it will average out diffusion coefficients.

The scale should be linear as very few DOSY show relevant signals that are orders of magnitude apart. Just ensure that the number of points used to render the diffusion dimension is at least 512 (see above). A small number will cause an artificial broadening in the diffusion dimension and will prevent representing well species with different diffusion coefficients. The use of exponential scales only serves to hide experimental errors and makes more difficult to distinguish species with close diffusion coefficients.

The minimum (DISPmin) and maximum diffusion ranges (DISPmax) should be chosen with care, as a large window makes more difficult to distinguish species with close diffusion coefficients (especially when combined with exponential representations).

Click here 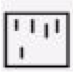 to start the fitting. The fitting used a mono-exponential equation. For sample with very good signal-to-noise ratio, biexponential fitting could be used, but these rarely work. This is the result:

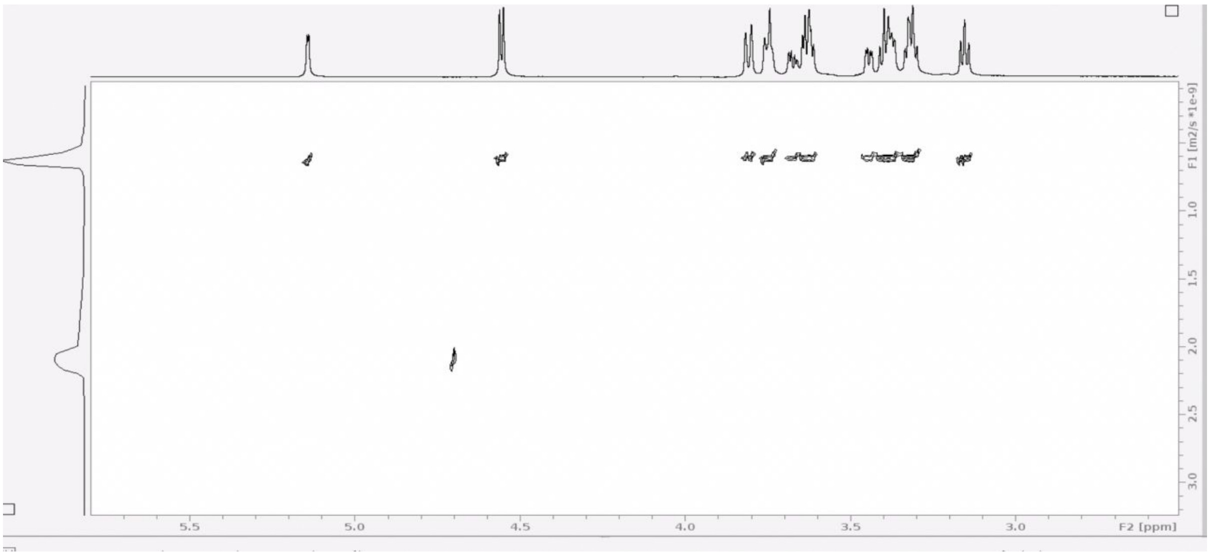

Figure S23. [1]H PROJECT-DOSY obtained following the previous instructions.

Multiplicity analysis using the T_2-filtered J-resolved PSYCHE-2DJ

The experiment is processed typing **xfb;apk2d;tilt;symj**. We would obtain pure shift 1D data projecting along the F2 dimension (the direct dimension), and the multiplicity by projecting to the F1 dimension. The latter works when only one multiplet is present on the window, for example, before projecting onto F2 we have:

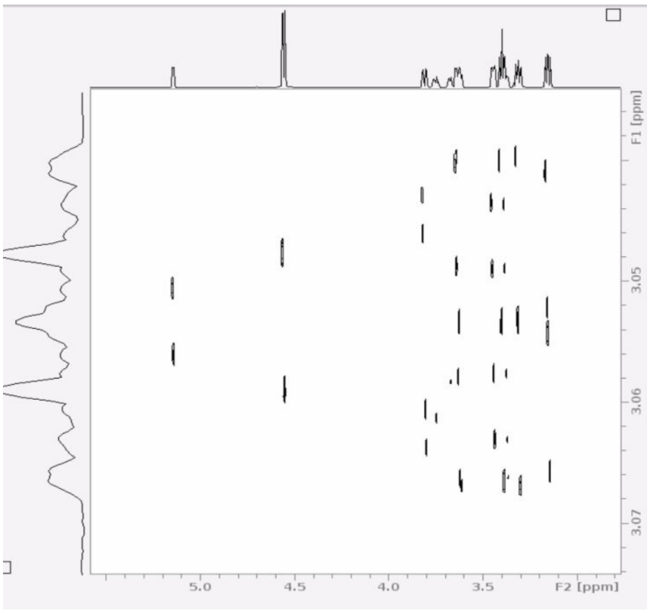

Figure S24. T_2-fitered 2DJ-PSYCHE-TSE of the polymer-containing sample. Notice that the broad signals are absent. This experiment allows the analysis of multiplicities in the indirect (F1) dimension. The following describes how to. This data has not been tilted, so the F2 projection is the equivalent to a T_2-filtered [1]H. Tilting will produce, by projection, a F2 T_2-filtered pure shift spectrum, as in the following figures.

The projection can be turned into a fid using an inverse Fourier transform.

After projecting and selecting signals:

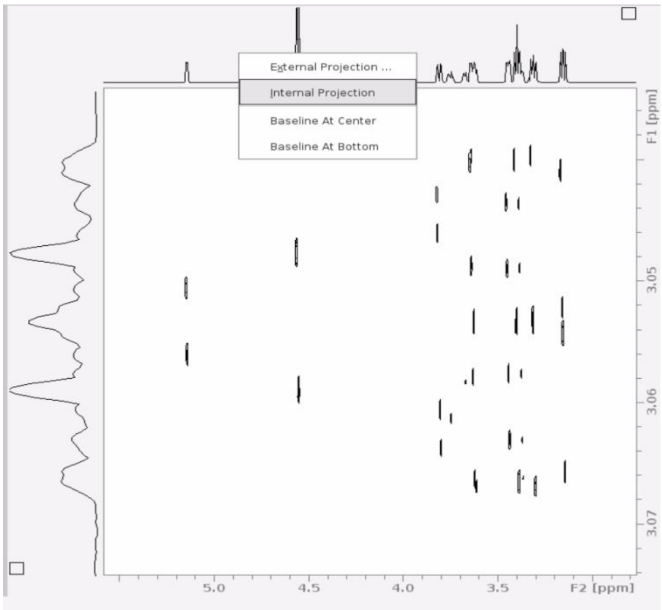

We have:

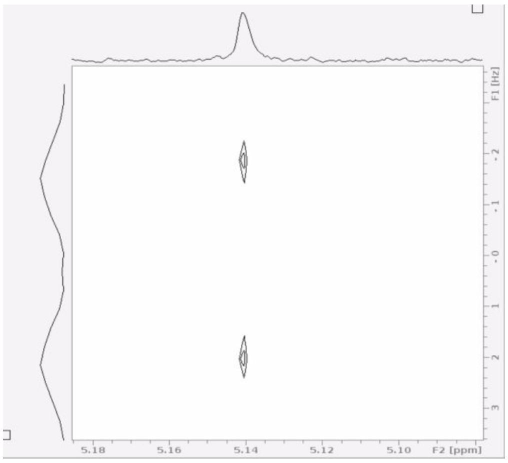

Figure S25. An example of how the T_2-fitered 2DJ-PSYCHE-TSE experiment can be used to produce pure shift data after tilting and how the F1 dimension allows multiplicity analysis.

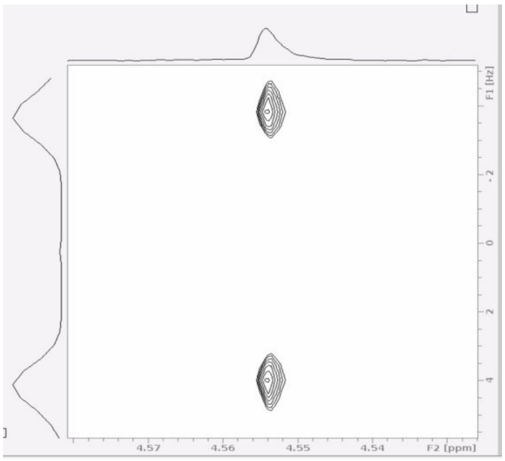

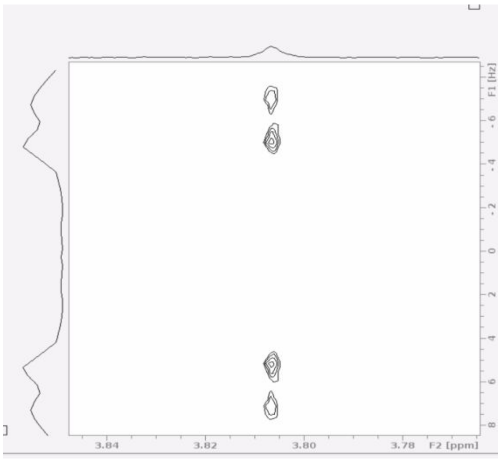
3.84
3.82
3.80
3.78
F2 [ppm]
F1 [Hz]

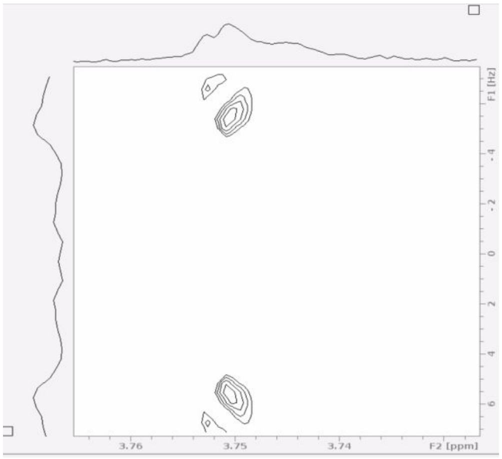
3.76
3.75
3.74
F2 [ppm]
F1 [Hz]

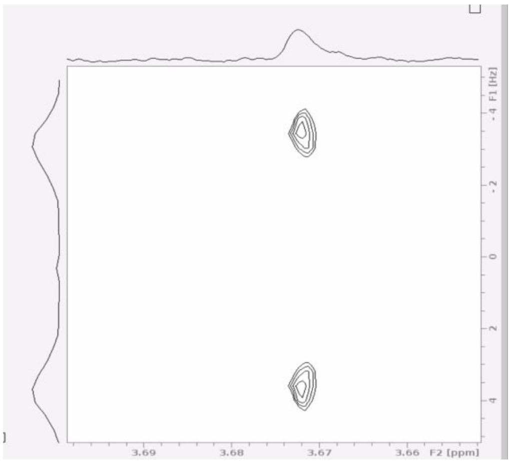
3.69
3.68
3.67
3.66
F2 [ppm]
F1 [Hz]

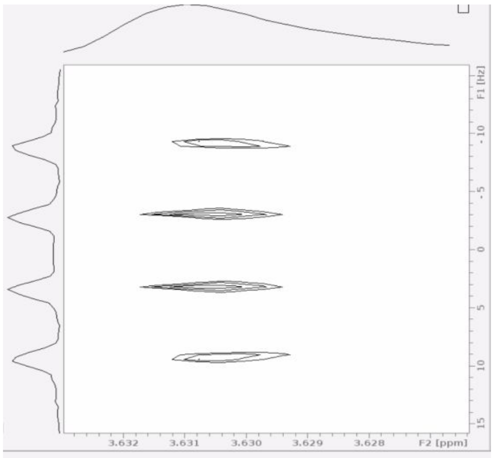
F1 [Hz]
-10
-5
0
5
10
15
3.632
3.631
3.630
3.629
3.628
F2 [ppm]

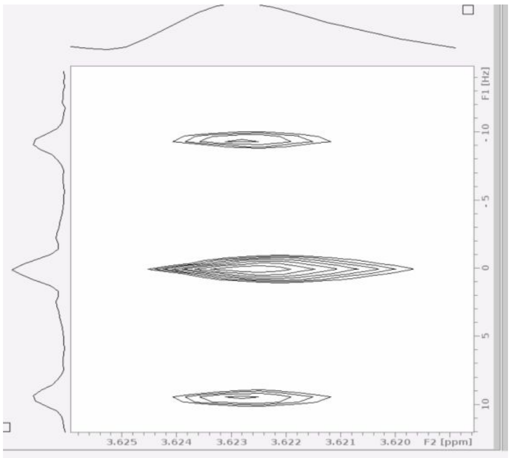
F1 [Hz]
-10
-5
0
5
10
3.625
3.624
3.623
3.622
3.621
3.620
F2 [ppm]

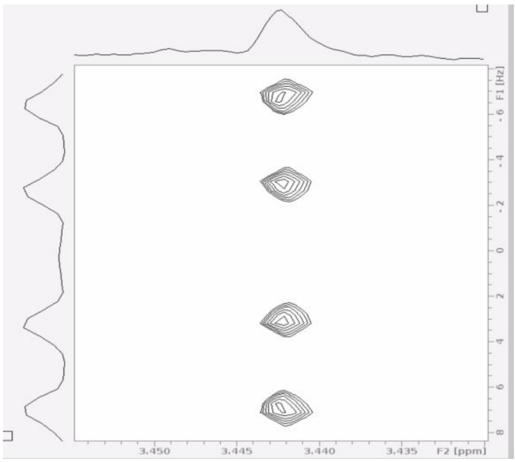
F1 [Hz]
-6
-4
-2
0
2
4
6
8
3.450
3.445
3.440
3.435
F2 [ppm]

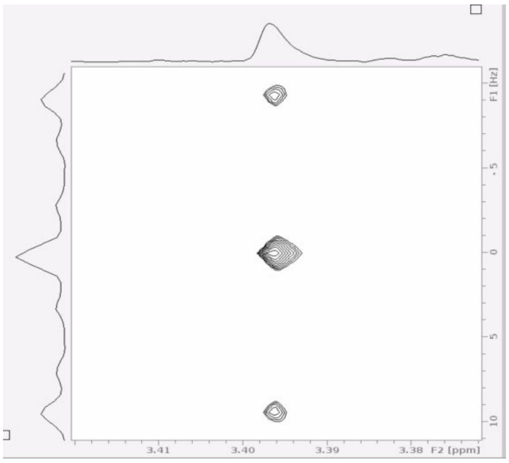

F1 [Hz]
3.41
3.40
3.39
3.38
F2 [ppm]

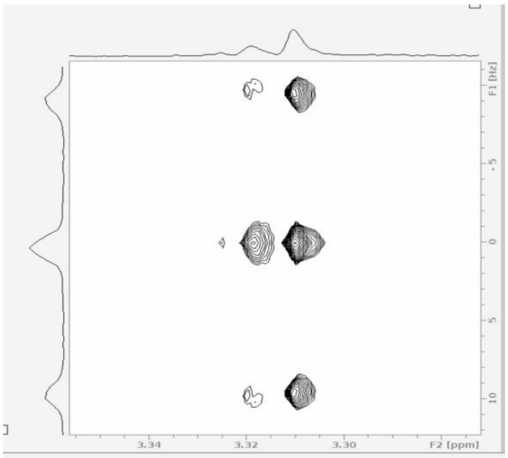

F1 [Hz]
3.34
3.32
3.30
F2 [ppm]

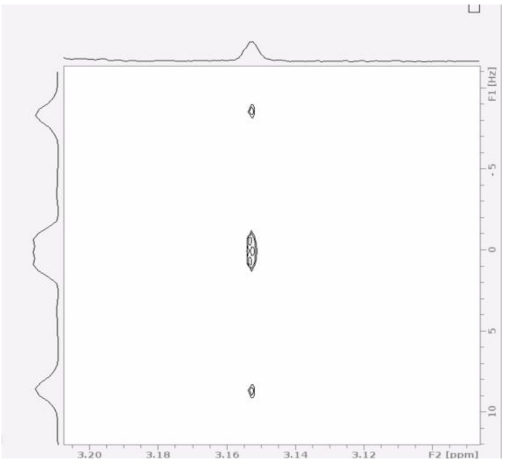

F1 [Hz]
3.20
3.18
3.16
3.14
3.12
F2 [ppm]

SECTION IV: additional evidence

Comparing PROJECT-DOSY results obtained using a sample of glucose in D_2O and a sample with just glucose.

Comparison between the T_2-filtered J-Resolved PSYCHE-TSE of glycerol dissolved in D2O with glycerol in the polymer sample.

Comparison between ^{1}H-NMR of glycerol dissolved in D_2O with glycerol in a water sample passed through the spin concentrator.

Use of WASTED-II to follow the degradation of the pollymer

Comparing PROJECT-DOSY results obtained using a sample of glucose in D_2O and a sample with just glucose.

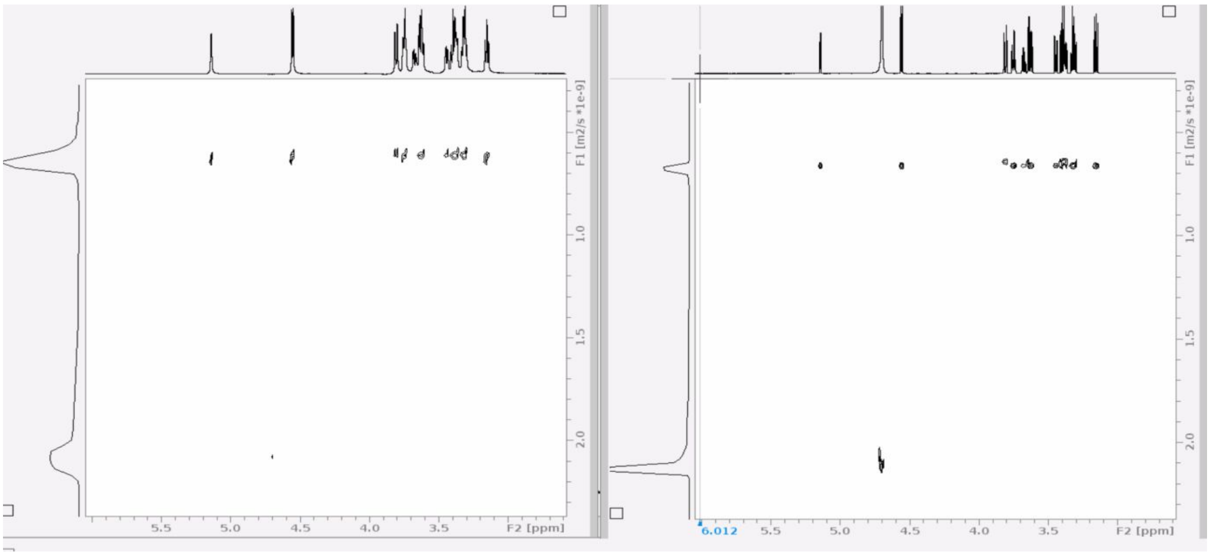

Figure S26. Left: PROJECT-DOSY of polymer-containing sample D_2O. The peaks show belong to glucose. The water signal was removed using the mathematical filter implemented in TopSpin. Right: PROJECT-DOSY of 10 mg of glucose in 600 µL of D_2O. The estimated diffusion coefficients are slightly small for the polymer sample (6.1 10^{-10} m^2 s^{-1}) than for the sample of pure glucose sample (6.6 10^{-10} m^2 s^{-1}) because the polymer sample has a higher viscosity and/or because glucose interacts weakly with the polymer.

Comparison between the T_2-filtered J-Resolved PSYCHE-TSE of glycerol dissolved in D_2O with glycerol in the polymer sample.

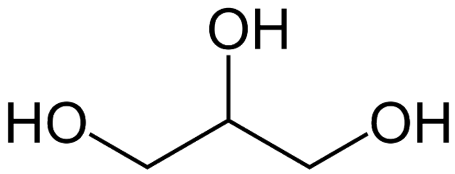

Glycerol

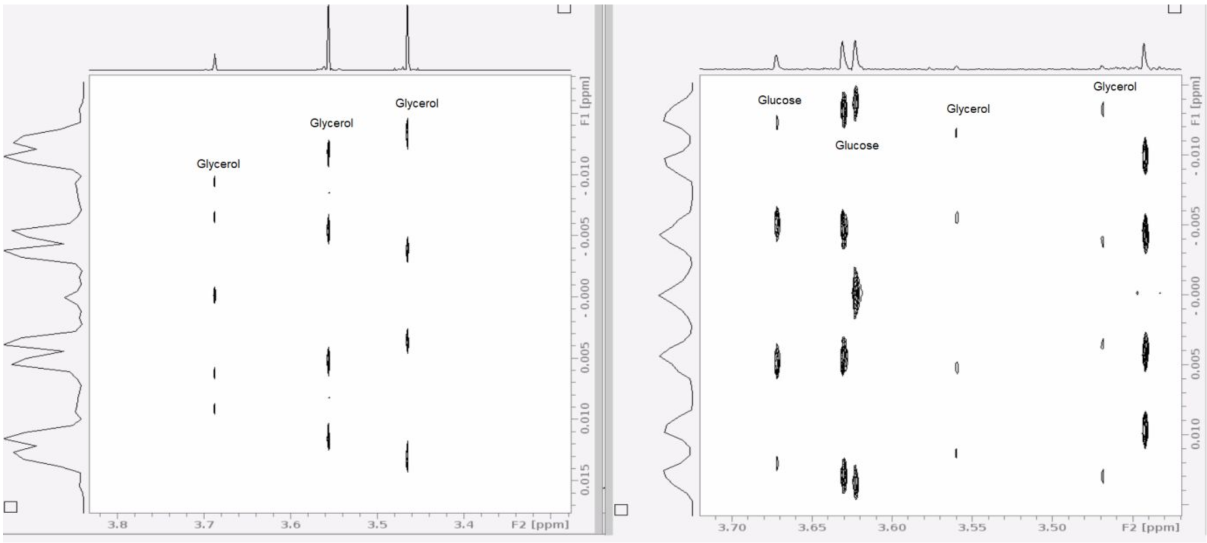

Figure S27. Left: J-resolved PSYCHE of glycerol in D_2O. Right: J-resolved PSYCHE of glycerol in the polymer sample. Note that the 3.69 ppm (CH) signal is absent is the right spectrum; this is due to the low concentration of glycerol in the sample and to the fact that that signal represents only one proton. Some signals from glucose also appear on the right spectrum.

Comparison between the ^{1}H-NMR spectrum of glycerol dissolved in D_2O and glycerol in a water that we passed through the spin concentrator.

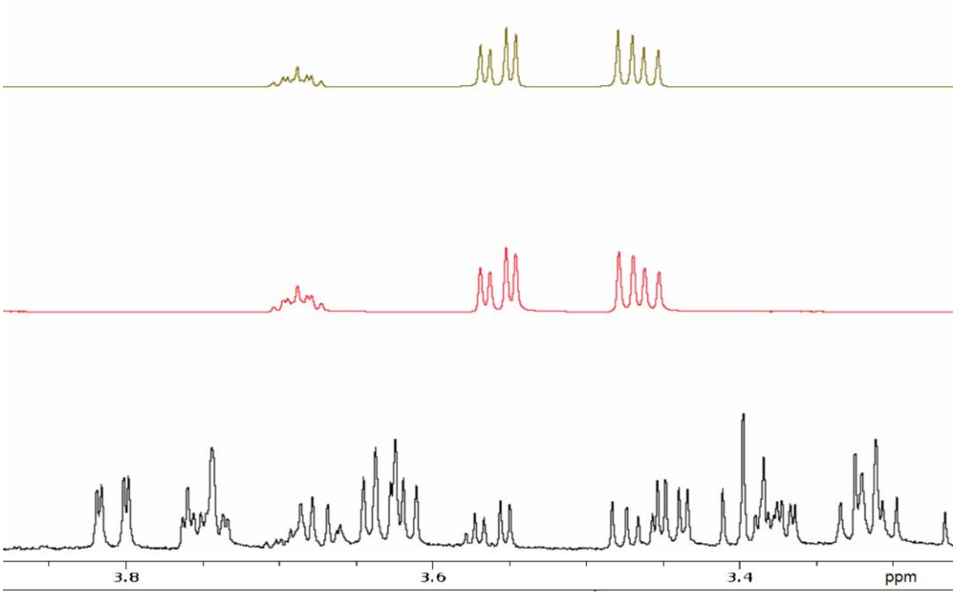

Figure S28. *Top:* 700 MHz ^{1}H NMR spectrum of pure glycerol dissolved in D_2O. Middle: 700 MHz ^{1}H NMR spectrum of the water that we passed through the spin-concentrator. Bottom: 700 MHz ^{1}H NMR spectrum of polymer sample containing glucose and glycerol. This confirmed that glycerol was present in the spin-concentrator.

Use of WASTED-II to monitor the degradation of the polymer

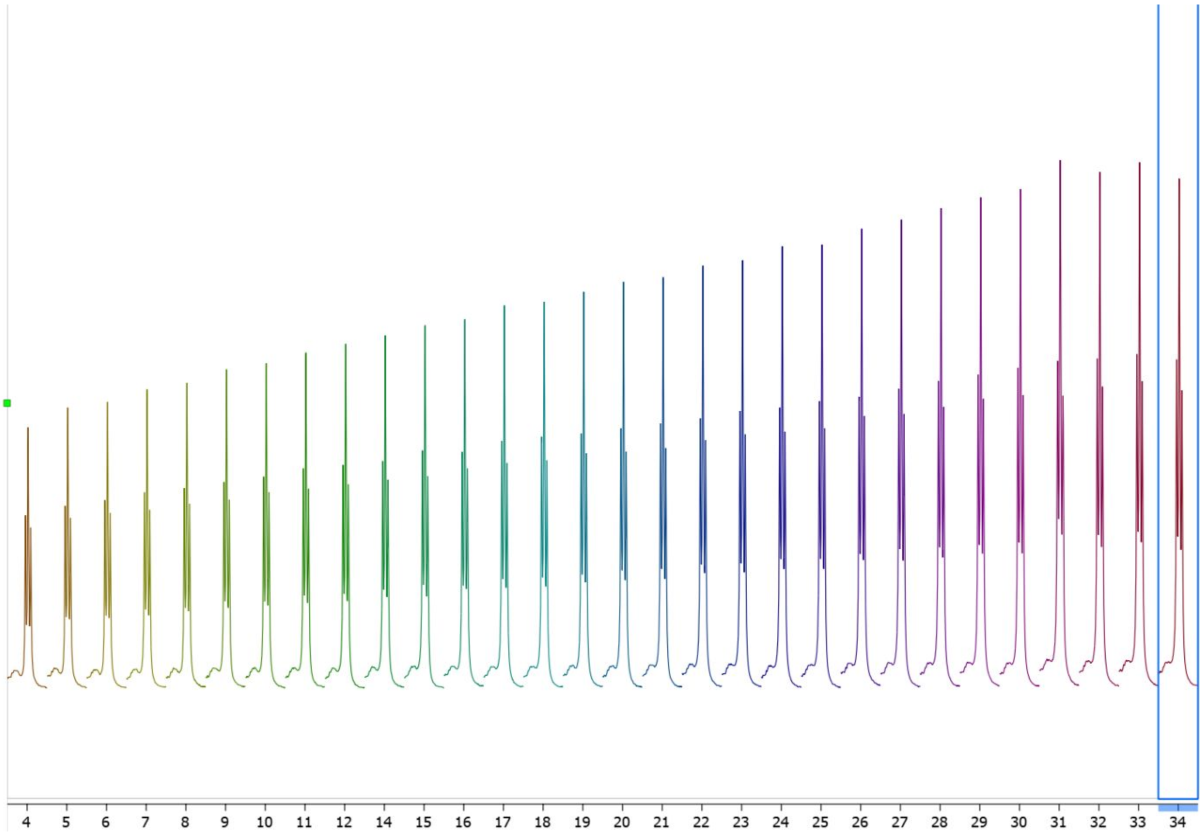

Figure S29. We used WASTED-II to monitor qualitatively the time it took for the sample to release glucose. One WASTED-II was acquired every hour and four minutes after the first nine hours. The figure plots the 3.15 ppm triplet as a function of time.

The sample precipitated around the 32th point, but it is likely that some precipitation occurred before, as the linewidth ($1/\pi T_2^*$) changed as it is apparent in the following stack where the same signal is plotted and normalized:

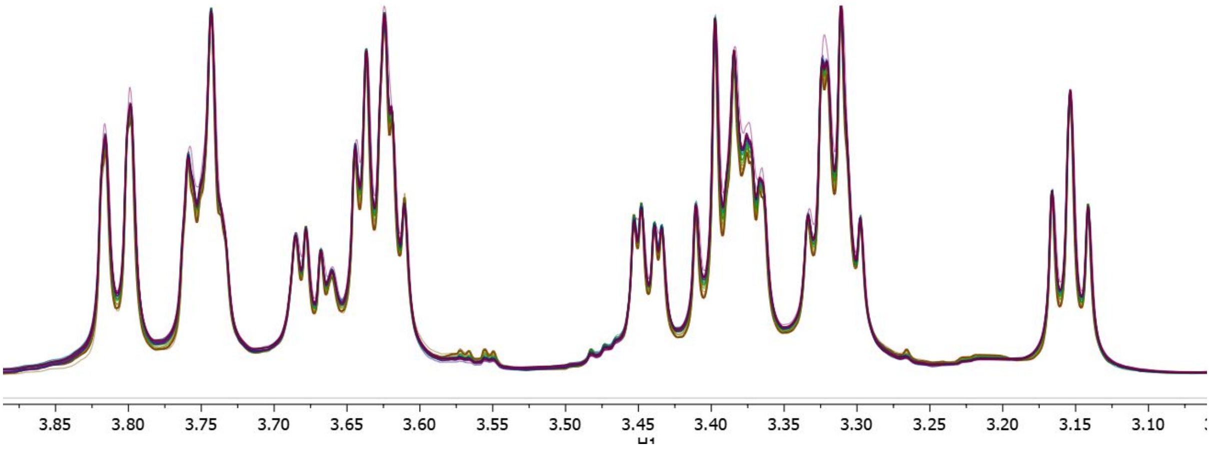

Figure S30. We superimposed all WASTED-II spectra to detect changes in viscosity and/or shims; such changes lead to signal broadening. Note the small but noticeable change in linewidth. This can also be appreciated plotting and normalizing the 3.15 ppm signal (next figure).

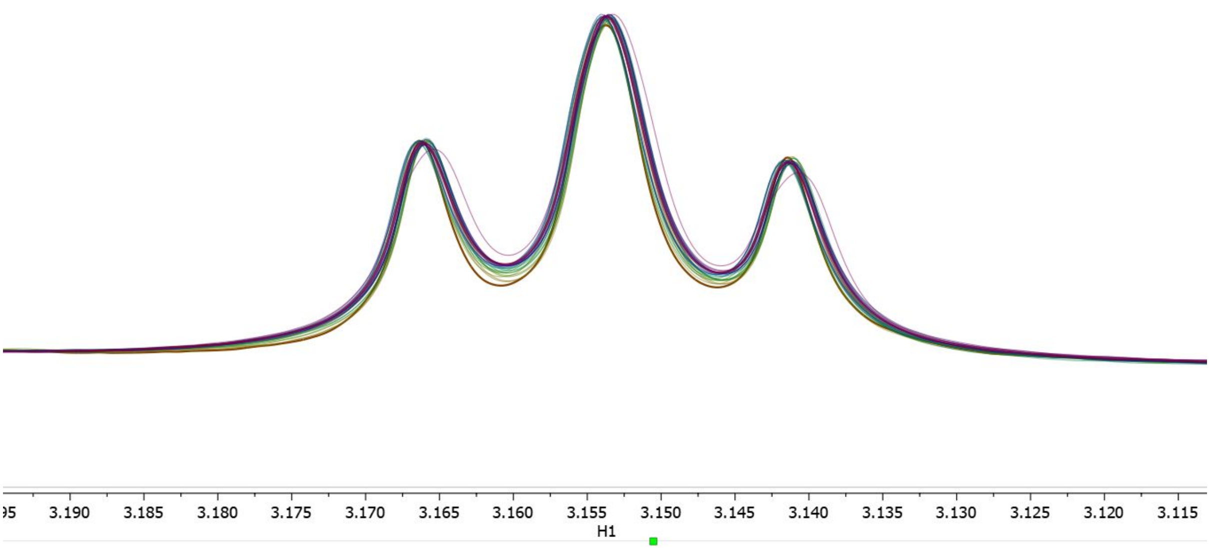

Figure S31. Superimposing all WASTED-II experiments shows a small-to-moderate change in viscosity that will be translated in losing some signal during the T_2 filter.

References

1. Lai, J. T.; Filla, D.; Shea, R. Functional Polymers from Novel Carboxyl-Terminated Trithiocarbonates as Highly Efficient RAFT Agents. Macromolecules. **2002**, *35*, 6754–6756. https://doi.org/10.1021/ma020362m.

2. Metz, N.; Theato, P. Controlled synthesis of poly(acetone oxime acrylate) as a new reactive polymer: Stimuli-responsive reactive copolymers. Eur. Polym. J. **2007**, *43*, 1202–1209. https://doi.org/10.1016/j.eurpolymj.2007.01.009.
